# Effects of dichlofluanid in tropical marine bivalves exposed to water and spiked sediments: an assessment of biomarker responses

**DOI:** 10.1007/s10646-026-03164-y

**Published:** 2026-10-01

**Authors:** Ana Carolina Feitosa Cruz, Paloma Kachel Gusso-Choueri, Guacira de Figueiredo Eufrásio Pauly, Bruno Galvão de Campos, Fernando César Perina, Ítalo Braga de Castro, Denis Moledo de Souza Abessa

**Affiliations:** 1 Núcleo de Estudos em Poluição e Ecotoxicologia Aquática (NEPEA), São Paulo State University - UNESP, Praça Infante Dom Henrique, s/n, São Vicente, 11330-900 SP Brazil; 2 Centre for Environmental and Marine Studies — CESAM, Department of Biology, Campus de Santiago, University of Aveiro, Aveiro, 3810-193 Portugal; 3 Santa Cecilia University – UNISANTA. Rua Oswaldo Cruz, 277. 11045-907. Santos, SP, Brazil; 4 Federal University of São Paulo – UNIFESP, Rua Carvalho de Mendonça, 144, Santos, 11070-100 SP Brazil; 5 Instituto nacional de identificação, quantificação, dispersão, riscos ambientais e mitigação da poluição por contaminantes emergentes em ambientes marinhos e costeiros (INCT CEMAR), 11330-900. São Vicente SP, Brazil

**Keywords:** Dichlofluanid, Antifouling, Biomarkers, Ecotoxicology, Organic matter

## Abstract

Antifouling paints have been used to control biofouling on submerged surfaces for decades. They contain biocidal compounds that may be released into the environment and harm aquatic ecosystems; however, their effects on tropical organisms are little known. Dichlofluanid, or N-dichlorofluoromethythio-N′,N′-dimethyl-N-phenylsulfamide (C_9_H_11_Cl_2_FN_2_O_2_S_2_), is a biocide present in antifouling paints and its toxicity to tropical marine organisms is poorly understood. This study aimed to evaluate the effects of dichlofluanid at the cellular and biochemical levels in bivalves exposed to seawater and sediment contaminated with this biocide, considering sediments with two different concentrations of organic matter. Aqueous-phase tests were performed using the mussel *Perna perna*, and tests with sediments were performed using the clam *Anomalocardia flexuosa.* Then, cellular and biochemical biomarkers were analyzed. The neutral red retention time assay (NRRT), a cellular biomarker, was assessed only in mussels, while biochemical biomarkers (DNA damage, LPO, GSH, and activities of GST, GPx, EROD, and AChE) were analyzed in both organisms. Particle size, calcium carbonate content, and organic matter analyses were also conducted on the sediments. At higher concentrations, the NRRT assay showed effects on mussel hemocytes, indicating a loss of lysosomal stability. Mussels also showed changes in the biochemical biomarkers of the digestive glands and gills. In clams exposed to sediment, adverse effects occurred in both organs and were more evident in animals exposed to sediments with higher levels of organic matter. Our study demonstrated that dichlofluanid can affect marine bivalves at realistic environmental concentrations and that organic matter may contribute to dichlofluanid exposure to clams.

**Supplementary information:**

The online version contains supplementary material available at https://doi.org/10.1007/s10646-026-03164-y.

## Highlights

Dichlofluanid affected lysosomes of the mussel *Perna perna* from 10 µg/L.

Dichlofluanid affected gills and digestive glands of *Perna perna* from 1 µg/L.

Dichlofluanid affected gills and digestive glands of *Anomalocardia flexuosa* from 1 ng/g.

Organically richer sediments induced worst effects in *Anomalocardia flexuosa*.

Effects of dichlofluanid in bivalves occurred at realistic environmental concentrations.

**Supplementary information:**

The online version contains supplementary material available at https://doi.org/10.1007/s10646-026-03164-y.

**Figure:**
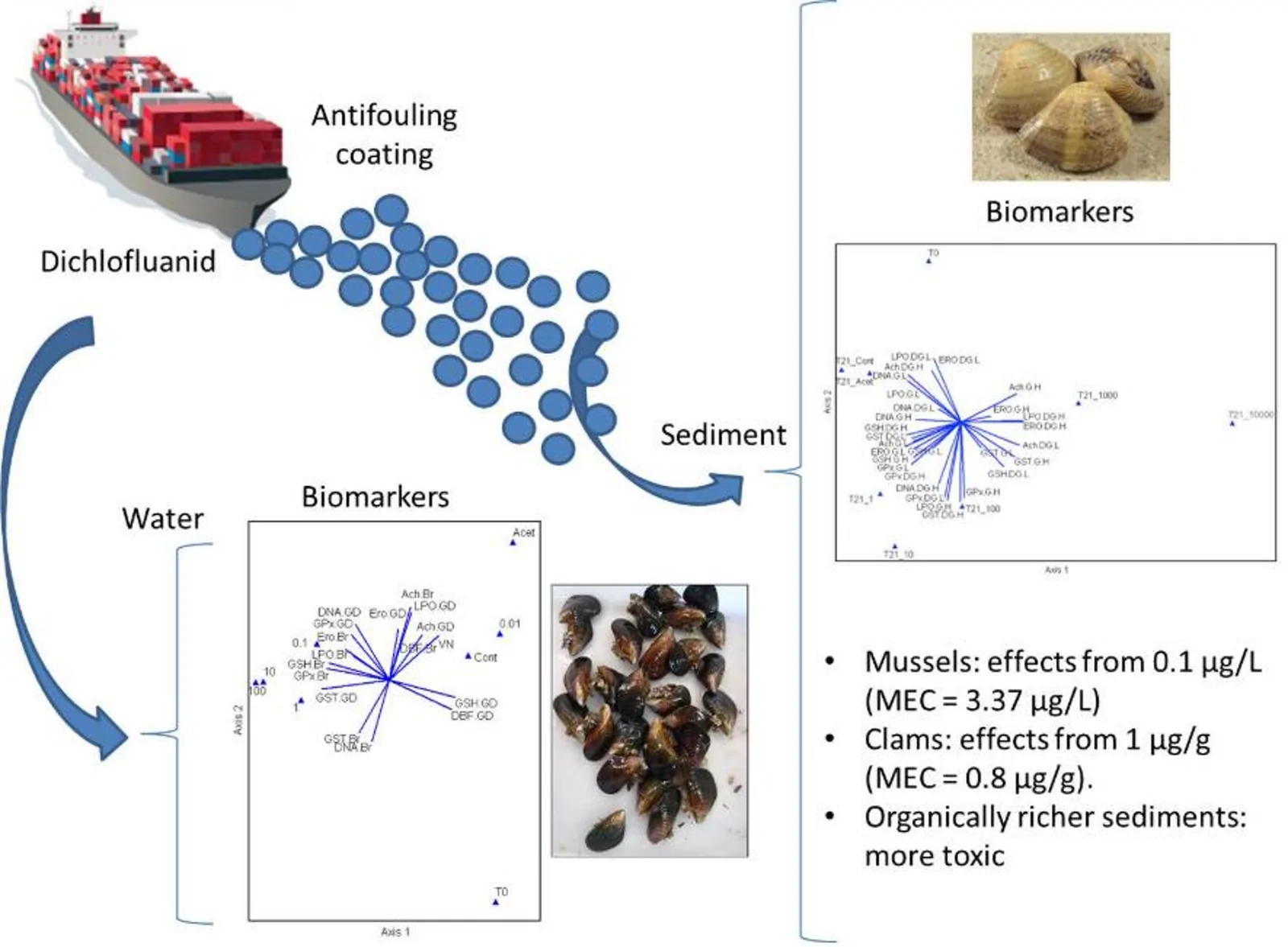

## Introduction

Biofouling is defined as the settlement of organisms on natural and anthropogenic hard surfaces submerged in water. Manmade structures affected by biofouling include oil platforms, pipelines, dam gates, aquaculture facilities, and small and large vessels. In commercial vessels, biofouling can increase fuel consumption by up to 40%. Biofouling accelerates hull corrosion, thereby increasing the cost of naval transport. Furthermore, organisms attached to ship hulls favor bioinvasion mechanisms throughout commercial routes worldwide (Hilliam et al. 2024). The introduction of non-indigenous species (NIS) has been associated with a range of environmental, economic, and cultural impacts, including threats to human health (Molnar et al. 2008). Food chain imbalances and extinction of native species have been associated with a rapid increase in international trade in recent decades (Diagne et al. 2021). Therefore, the International Maritime Organization (IMO) has recommended strategies to avoid or reduce marine invasions, including those aimed at preventing or inhibiting biofouling. Hence, antifouling coatings have been developed and widely used to protect ship and boat hulls (Almeida et al. 2007). Most protective systems are currently based on paints containing one or a combination of biocides aimed at inhibiting biofilm formation and larval settlement on submerged surfaces (Thomas and Brooks 2010).

Antifouling biocides, which include a range of organic and inorganic substances, are potentially harmful to aquatic ecosystems. The first commercial antifouling paints using copper and zinc oxides were replaced by those based on organotin compounds, such as tributyltin (TBT), because of their low effectiveness (Fernandez and Pinheiro 2007). Since then, TBT-based antifouling paints have been extensively used worldwide, mainly because of their high durability. However, they were banned by the Antifouling Systems Convention issued by the IMO because of their widespread toxic effects, including endocrine disruptors detected in ship and boat traffic areas (Dafforn et al. 2011; Evans et al. 2000). Organotins, and particularly TBT, were proved to induce imposex in gastropods, with severe implications to marine life (Abreu et al. 2020; Gibbs and Bryan 1994) Thus, a new set of non-metallic and metallic compounds has been used as active booster biocides in the current generation of antifouling paints, including cuprous oxides, copper and zinc pyrithione, Zineb, DCOIT, Irgarol, Diuron, dichlofluanid, and chlorothalonil (Castro et al. 2011; Paz-Villarraga et al. 2022; Almeida et al. 2025).

Due to the extensive use of these compounds, areas with intense ship and boat traffic, such as ports, marinas, shipyards, and navigation channels, are exposed to biocide contamination leached from these coatings. In fact, reports of booster biocide residues reaching the water column and other environmental compartments, such as sediments and biota, are frequent (Albanis et al. 2002; Almeida et al. 2023; Campos et al. 2022a; Castro et al. 2012; Gatidou et al. 2007; Hamwijk et al. 2005; Korkmaz et al. 2025; Vouvoulis et al. 2000; Mukhtar et al. 2026), as well as the environmental risks caused by these substances, which involve acute toxicity, reproductive effects in invertebrates, physiological impairment, and inhibition of photosynthesis in algae (Abreu et al. 2021; Campos et al. 2022a).

Dichlofluanid, or N-dichlorofluoromethythio-N′,N′-dimethyl-N-phenylsulfamide (C_9_H_11_Cl_2_FN_2_O_2_S_2_), is a booster biocide currently used in antifouling paints (Paz-Villarraga et al. 2022). It is a non-metallic organochlorine compound with low water solubility and the potential to accumulate and associate with particulate matter, as its n-octanol/water partition coefficient (log Kow), defined as the ratio of the concentration of a chemical in n-octanol and water, is 3.7 (Dafforn et al. 2011; Evans et al. 2000). Dichlofluanid is also used as a fungicide and can induce mutagenic and carcinogenic effects in nontarget marine invertebrates (Bellas 2006). However, studies on dichlofluanid toxicity in marine organisms are scarce, particularly in polar and tropical organisms (Campos et al. 2022a). Thus, further investigations are needed to evaluate the biological effects of dichlofluanid on tropical aquatic organisms, which are often more sensitive to contaminants, and provide information for its regulation at the global and local levels.

The use of biomarkers to assess biochemical, physiological, or histological responses induced by dichlofluanid exposure at the organism or “sub-organism” levels may help elucidate the potential early impacts associated with this substance (Walker et al. 2005). Traditionally, such approaches have used organisms such as fish and invertebrates as suitable biological models (Monteiro et al. 2024). Bivalve mollusks have great ecological and commercial importance and have been widely used to assess the bioaccumulation and effects of contaminants (Pereira et al. 2014; USEPA 1993). They are abundant and easy to collect, allowing the use of many individuals per experiment, and their sessile habits guarantee exposure to contaminants, thus they may indicate the conditions of their surrounding environment. Moreover, bivalves are filter-feeding animals that inhabit both hard- and soft-bottom substrates directly exposed to water and/or sediment matrices (Pereira et al. 2014; USEPA 1993). In this sense, the organic matter present in sediments can influence the exposure of burrowing bivalves to dichlofluanid because organic compounds tend to have a higher affinity for organic carbon (Haitzer et al. 1998; Jones and De Voogt 1999). The sorption of dichlofluanid onto organic matter may reduce the fraction dissolved in water but may favor the exposure of benthic organisms to this compound via the feeding route (i.e., ingestion of particulate matter).

This study aimed to assess the cellular and biochemical effects of dichlofluanid on two species of marine bivalves exposed to water and sediment contaminated with this biocide. Additionally, we evaluated the influence of sediment organic matter (OM) enrichment on dichlofluanid toxicity by exposing bivalves to sediments containing different OM concentrations. The influence of OM on the toxicity of antifouling compounds is still overlooked and requires studies on this topic. We hypothesized that dichlofluanid could induce cellular and biochemical alterations in marine bivalves and that the presence of OM in sediment would reduce dichlofluanid bioavailability by decreasing its solubility and consequently its effects on *Anomalocardia flexuosa*.

## Materials and methods

### Water exposure

To assess the biochemical and cellular effects of dichlofluanid dissolved in water, adult individuals of the brown mussel *Perna perna* (mean length = 6.69 ± 0.53 cm) were employed as biological models. This species was chosen due to its ecological relevance as a filter-feeding mussel and its widespread use as a sentinel species in ecotoxicological studies (Pereira et al. 2014). Specimens were sourced from an aquaculture farm located at Cocanha Beach, Caraguatatuba, São Paulo (23°34’40.0"S, 45°18’52.8"W) and subsequently transported to the laboratory, where they were acclimatized under controlled experimental conditions.

The experiment was conducted according to the protocols described by the USEPA (2002; 1993). The exposure treatments used five nominal dichlofluanid concentrations (0.01, 0.1, 1, 10.0, and 100 µg/L) in seawater; these concentrations include the range of measured levels in seawater, from not detectable to 3.37 µg/L (Campos et al. 2022a; Martínez and Barceló 2001). Negative controls containing only clean seawater (collected from a pristine coastal area) and acetone (co-solvent) diluted in seawater (0.05% concentration) were prepared. Three replicates were established in chambers conditions for 24 h with gentle aeration at 25 ± 2 °C and under a controlled photoperiod (12 h light: 12 h dark). Spiking with dichlofluanid was then carried out, and 12 healthy *P. perna* individuals were introduced into each replicate. The total exposure period was 96 h. Every 24 h, or whenever gamete release by the organisms was observed, the test solutions were renewed, and a new solution was prepared to ensure the maintenance of initial dichlofluanid concentrations and overall water quality. Numerous studies have shown that dichlofluanid tends to degrade rapidly in water, with a half-life of just a few hours, generating by-products, such as N-dimethyl-N-phenyl-sulphamide (DMSA) and dichloromethane aniline (Sakkas et al. 2001; Vouvoulis et al. 2000). Thus, while daily renewals of test solutions aimed to maintain nominal concentrations, the rapid degradation of dichlofluanid suggests that actual exposure tends to be dynamic, potentially involving degradation products in addition to the original compound. The daily water renewal somehow should reproduce this process that would occur in nature, where organisms are exposed to the original compound and its by-products.

After 48 and 96 h of exposure, five individuals from each replicate of the respective treatment (totaling 15 per treatment) were analyzed using the neutral red retention time assay (Lowe et al. 1995); the organisms analyzed at 48 h were removed from the experiment. In addition, organisms were sampled at the beginning of the experiment (T0 treatment) and analyzed to check the experiment effect on each biomarker. Throughout the test, physicochemical parameters such as pH, dissolved oxygen, salinity, temperature, and ammonia were monitored and maintained within acceptable ranges to prevent any interference with the experimental results. The first four parameters were measured using appropriate electrodes (Pro pH Meter – Line lab; D.O. Meter Hannah HI9146; Multiparameter AKSO AK88v2), and ammonia concentrations were determined using a colorimetric method, following the protocol described by Verdouw et al. (1978). At the end of the experiment, the remaining organisms (i.e., 5 individuals per replicate, or 15 per treatment) were euthanized, and their soft tissues (gills and digestive glands) were removed and stored at -80 °C for subsequent analysis of biochemical biomarkers.

### Sediment exposure

The experiment using spiked sediments used two types of sediments, each characterized by its organic matter (OM) content: one sediment had a low OM content (~ 4%), whereas the other had a higher OM content (~ 10%). Both sediments were collected from distinct locations in the Cananéia-Iguape Peruíbe Estuarine Complex, a protected area on the southern coast of São Paulo State. Low-OM sediment was collected from Arrozal (25°02.415’ S, 47°55.540’ W), and high-OM sediment was collected from Ariri (25°13.215’ S, 48°02.492’ W). Both sediments were spiked with dichlofluanid to assess potential differences in toxicity related to organic matter content. As in the aqueous solution assays, negative controls (dichlofluanid-free) and analytical blanks (acetone-containing sediments of both types) were prepared, as well as organisms from T0 were sampled. The dichlofluanid concentrations tested in the sediment were 1, 10, 100, 1000, and 10,000 ng/g, respectively. These concentrations include levels already reported in coastal sediments (~ 1 µg/g; Abreu et al. 2020) and toxic levels (Campos et al. 2022a).

The clam *Anomalocardia flexuosa* was used as the biological model. Adult individuals (mean length = 2.01 ± 0.21 cm) were collected from the Cananeia region, São Paulo (25°02.415’ S, 47°55.540’ W), transported to the laboratory, and acclimatized to the test conditions. The experiments were conducted as described by the USEPA (1993, 2002). Six replicates were prepared in glass chambers containing a sediment layer approximately 4 cm thick and 1.5 L of filtered seawater. The test system was maintained under equilibrium conditions with gentle aeration at 25 ± 2 °C. After a 24-h acclimation period, seven healthy *A. flexuosa* individuals were introduced into each replicate (totaling 42 per treatment). The tests were conducted under a controlled photoperiod (12 h light: 12 h dark), with continuous aeration and constant temperature (25 ± 2 °C) for 21 days. Throughout the experimental period, physicochemical parameters, including pH, salinity, temperature, dissolved oxygen, and ammonia, were monitored using the methodologies described previously for aqueous solution tests.

### Sedimentological analyses

The sediment grain size distribution was analyzed according to the protocol proposed by Mudroch and MacKnight (1994). Three aliquots (30 g) from each sample were dried in an oven at 60 °C for two days, washed through a 0.063 μm sieve to separate fine particles (silt and clay), and the sandy material retained on the sieve was dried again, sieved for 15 min in a RO-TAP shaker using sieves of different mesh sizes (Φ scale), and then weighed. The results were classified according to the Wentworth Scale (Wentworth 1922). The calcium carbonate (CaCO₃) content in each sample was measured using the method described by Hirota and Szyper (1975), which involved sample digestion with 5 N HCl for more than 24 h to remove CaCO_3_, followed by washing with distilled water and drying in an oven at 60 °C. The percentage of CaCO₃ in the sample was calculated from the observed weight loss. The organic matter (OM) content was estimated using the ignition method described by Luczak et al. (1997), in which aliquots of dry sediment were incinerated in a muffle furnace at 500 °C for 4 h. The percentage of OM was equal to the weight loss during ignition.

### Sediment spiking

Sediment spiking was performed according to the protocols described by ASTM (2008). Five dichlofluanid concentrations were used in the tests: 1, 10, 100, 1000, and 10,000 µg/g, respectively. To prepare each, the respective amounts of dichlofluanid were added to the sediment based on the dry weight% of the sediment (inversely to the wet weight). The moist sediment was partitioned into aliquots, and different concentrations of dichlofluanid were added. The analytical blank (clean sediment containing acetone) was subjected to the same procedure, and the control (clean) sediment consisted of natural sediments. All sediment samples were agitated for 15 min using a device that provided multidirectional rotation in a hermetically sealed glass container. After mixing, the contaminated sediments were stored in the dark at 4 °C for 72 h to allow equilibrium to be established between the contaminant, interstitial water, and the solid sediment phase. Subsequently, the sediments were distributed into the test chambers for exposure experiments. Comprehensive descriptions of the sediment spiking methodology and analytical confirmation of dichlofluanid concentrations in the tested sediments were provided by Campos et al. (2024). Dichlofluanid levels were determined using gas chromatography-mass coupled with Mass spectrometry (GC-MS).

### Biomarkers analyses

#### Neutral red retention time (NRRT)

The analysis of Neutral Red Retention Time (NRRT) in hemocyte lysosomes followed the protocol described by Lowe et al. (1995). This biomarker was assessed only in experiments involving exposure to water. Prior to the assay, the glass slides were pre-treated with a poly L-lysine solution to facilitate hemocyte adhesion to the slides. Hemolymph samples (0.5 mL) were extracted from the adductor muscle of the mussels using a hypodermic syringe containing physiological solution (0.5 mL). After 20 min, 40 µL of cell suspension was placed on each slide. The slides with hemocytes were kept in a dark and humid chamber for 15 min. Subsequently, 40 µL of Neutral Red solution was added to each slide. After 15 min, the coverslips were placed on the samples. The slides were then examined under a microscope at 15-minute intervals during the first hour and subsequently at every 30 min for a maximum of 120 min. The cells were analyzed to determine the retention time of neutral red in the lysosomes. The NRRT was obtained by estimating the proportion of cells in which lysosomal content leaked into the cytosol. When 50% or more of the cells on a glass slide exhibited such leakage, the organisms were considered physiologically stressed.

#### Biochemical biomarkers

The gills and digestive glands of both *P. perna* and *A. flexuosa* were kept on ice and homogenized in Tris-HCl buffer (50 mM TRIS; 1 mM EDTA; 1 mM DTT; 50 mM sucrose; 150 mM KCl; 100 mM PMSF; pH 7.6). Following homogenization, aliquots were separated for total protein (TP), DNA damage, and lipid peroxidation (LPO) analyses. The homogenates were centrifuged at 12,000 × g for 20 min at 4 °C, and additional aliquots were collected for the analysis of TP, glutathione S-transferase (GST), and glutathione peroxidase (GPx) activity, as well as for the quantification of non-protein thiols (GSH), acetylcholinesterase (AChE) activity, ethoxyresorufin-O-deethylase (EROD), and dibenzylfluorescein (DBF). All biomarker analyses were performed using a Biotek microplate reader (model Synergy HT).

Total proteins were quantified using the Bradford method (Bradford 1976), and this content was used to normalize all biomarkers data. DNA damage was assessed using the alkaline precipitation assay proposed by Olive (1988), with fluorometric DNA quantification (λex 360 nm; λem 450 nm) (Gagné et al. 2006). The results were expressed as µg of DNA per milligram of protein. LPO levels were determined by measuring thiobarbituric acid reactive substances (TBARS) using fluorescence detection (λex 532 nm; λem 556 nm), as described by Wills (1987). The concentration of peroxidized lipids was expressed as µM TBARS per mg of protein. AChE activity was measured at 412 nm using the colorimetric method (Ellman et al. 1961), and the results were expressed as µmol DTNB per minute per milligram of protein. GSH levels were determined according to the method described by Sedlak and Lindsay (1968), as follows. GSH levels were measured spectrophotometrically at 415 nm and expressed as nanomoles of glutathione per milligram of protein (nmol/mg). GPx activity was determined spectrophotometrically at 340 nm, as described by Sies et al. (1979), and the results were expressed as nmol per minute per mg of protein. GST activity was measured at 340 nm and 25 °C using a microplate reader (Habig et al. 1974). The results were expressed as optical density (OD) per minute per milligram of protein. EROD activity was evaluated using a modified method described by Gagné and Blaise (1993), and the results were expressed as pmol per minute per mg of protein. Dibenzylfluorescein (DBF) activity was determined according to Gagné et al. (2007), using fluorescence (λex 485 nm and λem 516 nm), and results were expressed as nmol per minute per mg of protein.

#### Statistical and exploratory analyses

The results from the NRRT assay were first checked for normality and homoscedasticity (Shapiro-Wilks test and Hartley test, respectively), then analyzed using one-way ANOVA with Dunnett’s post hoc test to detect significant differences in NRRT between the dichlofluanid concentrations, analytical blank (acetone), and control. The results obtained for biomarkers were first assessed for normality using the Kolmogorov–Smirnov test and variance homogeneity using the Hartley test, followed by one-way ANOVA with Dunnett’s post hoc test (or an equivalent non-parametric test when necessary) to determine significant differences among dichlofluanid concentrations, the blank (acetone), and the control in the water exposure tests. For the sediment exposure experiments using matrices with different organic matter contents, a two-way ANOVA with Bonferroni’s post hoc test (or PERMANOVA for non-parametric data) was conducted to assess the effects of dichlofluanid concentration, sediment type, and potential interactions between them. The results from the subchronic exposure tests (biomarkers) were further integrated using Principal Component Analysis (PCA) to identify patterns and potential groupings based on exposure conditions. For the sediment dataset, PERMANOVA was performed using the studied biomarkers (GSH, GST, GPx, EROD, AChE, LPO, and DNA damage).

## Results and discussion

### Water exposure – *Perna perna*

The physicochemical parameters of seawater at the *Perna perna* cultivation site and those recorded in the test solutions during the experiments are presented in the Supplementary Material (Tables S1 and S2). During the tests, the mortality rates remained below 20% of the total number of exposed organisms (Figure S1).

### Neutral red retention time – *Perna perna*

**Fig. 1:**
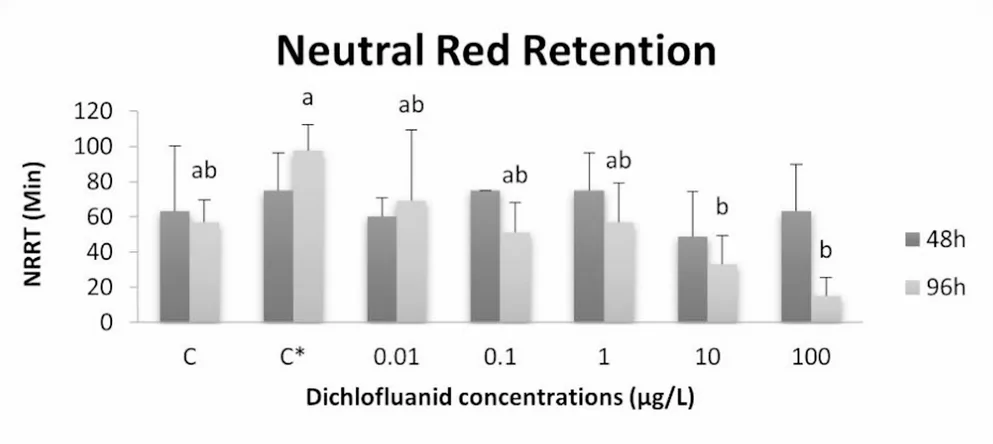
Neutral Red Retention Time (NRRT) in *Perna perna* hemocytes after 48 and 96 h of exposure to different concentrations of dichlofluanid, expressed in µg/L (ANOVA, *p* < 0.0001; *n* = 15). Letters indicate statistically significant differences from the acetone control (*p* < 0.05). Error bars represent the standard deviation. C = control; C* = acetone control 0.05%

Figure 1 presents the Neutral Red Retention Times (NRRT) after 48 and 96 h of exposure to dichlofluanid. At the initial time point, the organisms exhibited a mean retention time of 40.5 min. After 96 h, NRRT was significantly reduced (*p* < 0.05) in mussels exposed to 10 µg/L (mean NRRT = 33 min) and 100 µg/L (mean NRRT = 15 min) compared to the acetone control group (mean NRRT = 97.5 min). No significant differences in NRRT were observed after 48 h of exposure. Hagger et al. (2005) reported that for the mussel *Mytilus edulis*, increased cytotoxicity induced by TBT using the Neutral Red assay occurred at concentrations above 0.5 µg/L and at shorter retention times than those observed in the present study. These findings suggest that dichlofluanid may exhibit lower cytotoxicity than TBT, a compound used in antifouling coatings that has been banned in several countries.

Lysosomes are organelles responsible for handling tissue nutrition and repair, encompassing the main cellular structure for the sequestration and detoxification of organic compounds, as reported in previous studies (Lowe et al. 1995). Under chemical stress, lysosomal membranes are damaged, and the leakage of lysosomal contents into the cytoplasm often occurs concurrently. Neutral red retention is minimally affected by natural factors and is predominantly influenced by contaminants (Ringwood et al. 1998). Diclofluanid was reported to induce cytotoxicity in rat hepatocytes (Suzuki et al. 2004) corroborating the results of the present study. In our study, the significant responses observed for NRRT at higher dichlofluanid concentrations are concerning because lysosomal membrane stability and integrity have been widely used for the early detection of toxic compound effects (Repetto et al. 2008). NRRT serves as an important indicator of cellular health, and any impairment in this parameter may indicate early signs of more severe adverse effects across other levels of biological organization (Dailianis et al. 2003) to effects observable at the population and community levels.

### Biochemical biomarkers – *Perna perna*

**Fig. 2:**
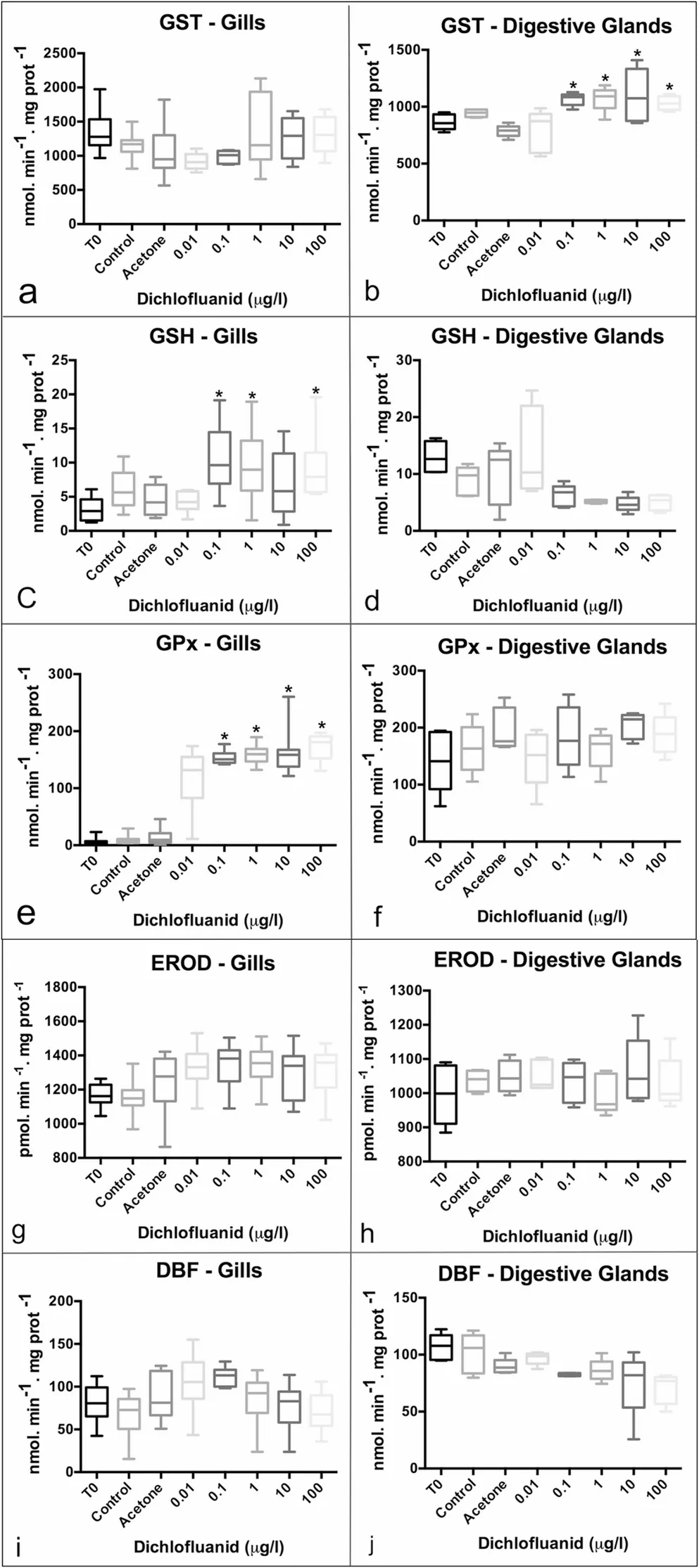
Exposure biomarker responses in the gills and digestive glands of the mussel *Perna perna* exposed to Dichlofluanid through water (*n* = 15 per treatment). Data are presented as box plots, with the boxes indicating the 25th and 75th percentiles; the line within each box represents the median value. Asterisks (*) above the data indicate statistically significant differences compared to the acetone control (*p* ≤ 0.05). Figure 2.a. ANOVA, *p* = 0.0103; Fig. 2.b. ANOVA, *p* = 0.0005; Fig. 2.c. ANOVA, *p* = 0.0002; Fig. 2.d. ANOVA, *p* = 0.0013; Fig. 2.e.Kruskal–Wallis, *p* < 0.0001; Fig. 2.f. ANOVA, *p* = 0.2299; Fig. 2.g. ANOVA, *p* = 0.0028; Fig. 2.h.ANOVA, *p* = 0.7243; Fig. 2.i.ANOVA, *p* = 0.0005; Fig. 2.j. ANOVA, *p* = 0.0043

Figure 2 presents the results for the exposure biomarkers. The induction of GST activity in the digestive glands was observed at the highest dichlofluanid concentrations, as well as increased levels of GSH and GPx activity in the gills at these concentrations. This induction of the antioxidant defense system has been previously reported for biocides used in antifouling paints, such as TBT, Irgarol, and Diuron. Cells have different mechanisms to cope with oxidative stress, either by repairing damage or reducing its occurrence through enzymatic and non-enzymatic antioxidant pathways. In this context, the action of enzymatic antioxidants, such as GST and GPx, can neutralize oxidative stress (Sutcu et al. 2007). However, responses were distinct between tissues, since GST activity in gills was not altered, as well as GSH quantities and GPx activity in digestive glands. Other measured exposure biomarkers were not altered in both tissues, such as EROD and DBF.

**Fig. 3:**
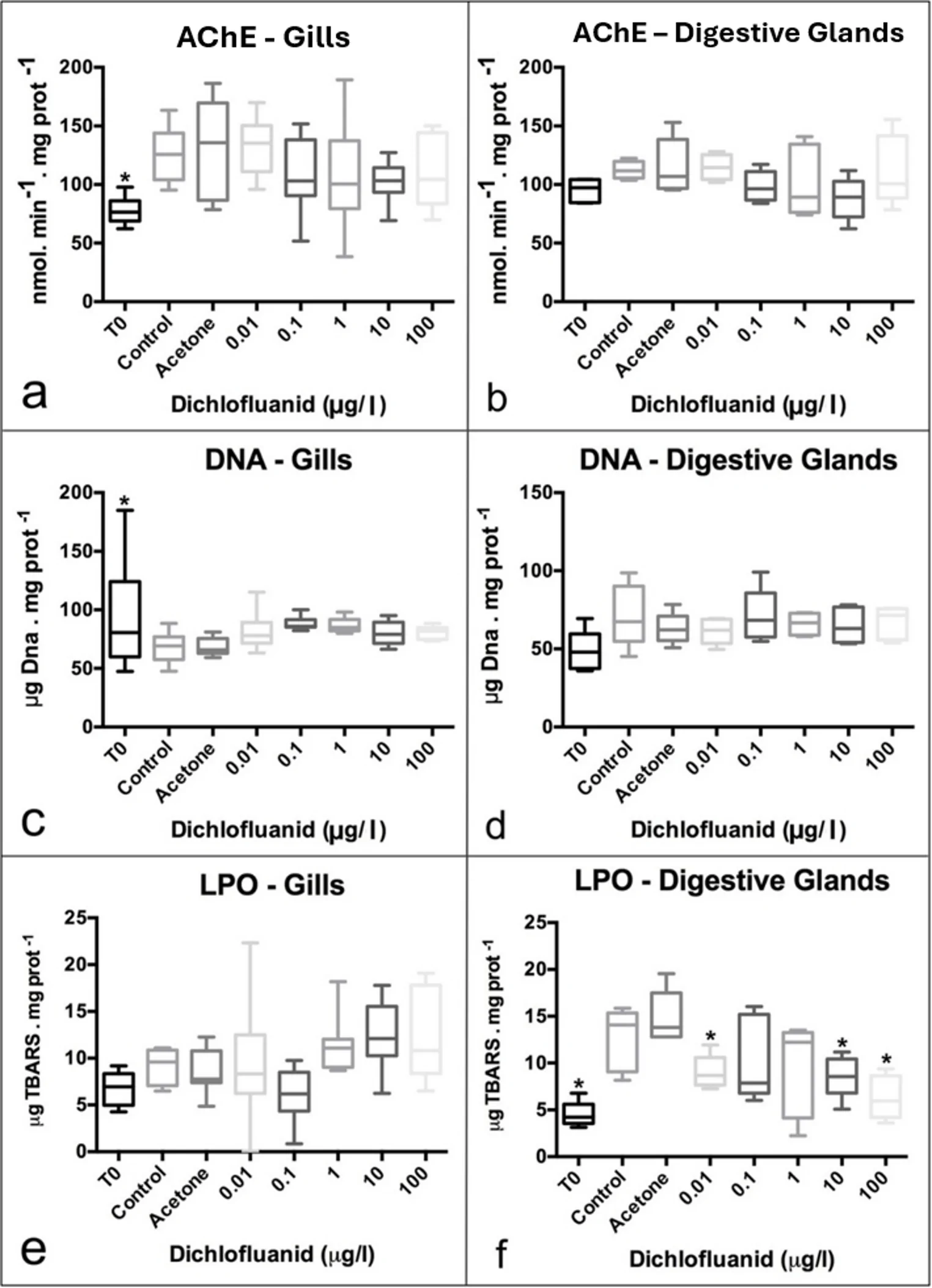
Effect biomarker responses in the gills and digestive glands of the mussel *Perna perna* exposed to Dichlofluanid through water (*n* = 15 per treatment). Data are presented as box plots, with the boxes representing the 25th and 75th percentiles. The line within each box indicates the median value. Asterisks (*) above the data denote statistically significant differences compared to the acetone control (*p* ≤ 0.05). Figure 3.a. ANOVA, *p* = 0.0027; Fig. 3.b. ANOVA, *p* = 0.3107; Fig. 3.c. ANOVA, *p* = 0.0296; Fig. 3.d. ANOVA, *p* = 0.1856; Fig. 3.e. ANOVA, *p* = 0.0002; Fig. 3.f. ANOVA, *p* = 0.0004

Regarding the biomarkers of effect, lipid peroxidation (LPO) levels in the digestive glands were lower than those observed in the control groups (Fig. 3), and no significant effects were observed in gills. Moreover, AChE activity and DNA damage were not affected in both gills and digestive glands. This observation, alongside the induction of detoxification enzymes, suggests that activated detoxification mechanisms may have minimized oxidative damage to cellular membranes in *P. perna*.

### Principal component analysis (PCA) – *Perna perna*

**Fig. 4:**
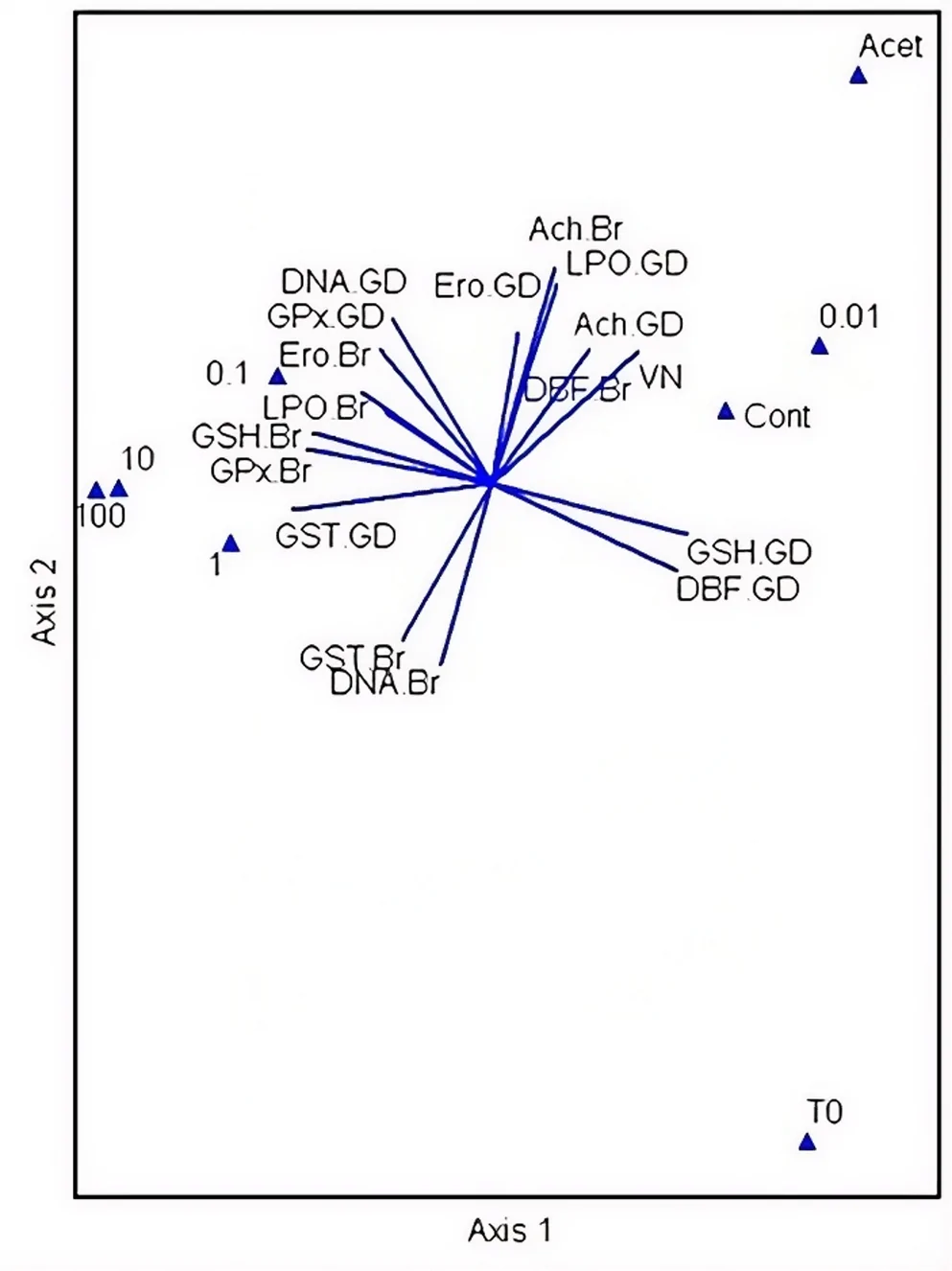
Projection of variables on a plane formed by factors 1 and 2 resulting of Principal Components Analysis (PCA) integrating all biomarkers analyzed in *Perna perna* exposed to various dichlofluanid concentrations in water. Br = Gills; GD = Digestive Gland; VN = Neutral Red Retention Assay

The PCA results for the water-based exposure tests are presented in Tables S3 and S4 and Fig. 4. The first two principal components (PCs) explained 64.54% of the total variance in the dataset (Table S3). The PCA revealed that for PC1, the seawater control, acetone blank, initial time point (T0), and lowest dichlofluanid concentration (0.01 µg/L) clustered together, whereas the other tested concentrations (0.1, 1, 10, and 100 µg/L) formed a second group (Table S3, Fig. 4). Regarding PC2, the control and analytical blank clustered with the two lowest dichlofluanid concentrations (0.01 and 0.1 µg/L), whereas the higher concentrations (1, 10, and 100 µg/L) were separated. T0 appeared to be isolated from the other treatments (Table S3, Fig. 4).

GPx and GSH in the gills and GPx and GST in the digestive glands were positively correlated with PC1, whereas DBF and GSH in the digestive glands presented negative correlations with PC1 (Table S3). These findings suggest the activation of detoxification mechanisms in both the gills and digestive glands, which is also reflected in the individual biomarker plots. Although no statistically significant differences were detected, the plots for the digestive glands showed a decreasing trend in GSH and DBF levels (Fig. 2d and j, respectively). Moreover, a relationship between enzymatic responses and the neutral red retention assay was observed. These effects were associated with intermediate and high dichlofluanid concentrations, although they did not result in measurable biochemical damage (i.e., lipid peroxidation and DNA damage). However, membrane-destabilizing effects were evident at higher concentrations, as demonstrated by significant lysosomal membrane destabilization in the NRRT assay. As this is a biomarker of effect, the overall data suggest that detoxification pathways were not fully effective, leading to lysosomal membrane damage, particularly at higher concentrations. Regarding PC2, negative correlations were found between GST and DNA damage in the gills, whereas positive correlations were observed between GPx and EROD, as well as between DNA damage, EROD, lipid peroxidation (LPO), and AChE in the digestive glands. This PC appears to have grouped variables that, when analyzed individually, did not show significant effects and thus did not contribute to a clear overall response in biomarker data. This indicates that, while PCA is a powerful and useful tool to integrate complex datasets, the biological significance of components composed of non-significant individual biomarkers should be interpreted with caution. Additionally, as this experiment was based on nominal concentrations, yet renewed at every 24 h, there is an uncertainty regarding the effects observed.

In the projection of variables relative to the two factors extracted by PCA, the treatments were grouped into three clusters plotted on the plane formed by PCs 1 and 2 (Fig. 4). T0 was isolated from the other samples. One group consisted of the control, acetone blank, and the lowest concentration (0.01 µg/L), whereas the other group contained intermediate and higher dichlofluanid concentrations (0.1, 1, 10, and 100 µg/L) (Fig. 4). These data suggest a metabolic effect in animals exposed to dichlofluanid at concentrations above 0.1 µg/L.

The analyses of biomarkers, either individually or in multivariate analyses, showed that dichlofluanid may induce significant alterations in antioxidant processes, as observed in the biomarkers directly involved with glutathione (GSH, GST, GPx). This can be explained by the mechanisms of action of dichlofluanid and its primary metabolite, DMSA (N’-dimethyl-N-phenyl-sulfamide). It is a multisite herbicide and fungicide, and its primary mode of action involves the reactions of dichlofluanid with thiols within the cells of the target organisms (Hamwijk et al. 2005). Thus, dichlofluanid is a reactive compound with sulfhydryl groups present in glutathione (Yamano and Morita 1995). This study demonstrated an increase in GPx activity and GSH production in the gills, as the primary exposure route in bivalves occurs via the gills. This increase in GPx activity and GSH levels may be directly related to the biotransformation processes of dichlofluanid or the potential reactive oxygen species generated in the presence of xenobiotics (Keppler and Ringwood 2001). Biocides such as dichlofluanid and DMSA have pro-oxidant properties that cause imbalances between GSH and GSSG, leading to several other cellular effects, including an increase in reactive oxygen species (ROS), which are eliminated by antioxidant enzymes such as GPx (Lushchak et al. 2018). In the digestive glands, GST activity and LPO levels increased at higher dichlofluanid concentrations, suggesting that the antioxidant system was limited and unable to avoid oxidative stress. The effects observed in the digestive glands suggest that dichlofluanid is incorporated by bivalves and transported to the glands, where detoxification processes occur. Digestive glands have been shown to be highly sensitive to xenobiotics in some bivalve species (Ramos-Gómez et al. 2011; Zhang et al. 2010).

Considering all the analyses performed when exposing bivalves to dichlofluanid via water, some effects were observed in both the gills and digestive glands, particularly at higher concentrations. However, no DNA damage or neurotoxicity was observed, and the effects on the lysosomal membranes of hemocytes were noted at higher concentrations, corroborating that the toxic effects of dichlofluanid are caused by oxidative stress, as lysosomal membranes have altered permeability due to ROS (Lowe et al. 1995). Thus, our results suggest that the effects of dichlofluanid are more closely related to membrane destabilization due to increased ROS levels. Studies have shown that bivalve hemocytes can be affected by biocides used as antifouling agents, such as DCOIT (Campos et al. 2022b) and chlorothalonil (Morais et al. 2023). Moreover, in ascidians exposed to dichlofluanid in concentrations ranging from 3.33 to 33.3 µg/L, hemocytes exhibited several alterations, such as apoptosis, cell shrinkage, reduced motility, and phagocytic activity (Cima and Varello 2020).

### Sediment exposure – *Anomalocardia flexuosa*

#### Sedimentological analyzes

Table 1 presents some sediment properties, including grain size distribution, calcium carbonate (CaCO₃) content, organic matter (OM) percentage, and wet weight. Both sediments used in the experiment consisted mainly of very fine sands mixed with mud and fine sands, as well as smaller quantities of other textural fractions, indicating that potential variations in toxicity are unlikely to be attributed to differences in sediment texture. The sediment collected from Ariri exhibited a higher OM content (10.4%) than that from Arrozal, which contained approximately 4% OM. As for CaCO₃ concentrations, no substantial differences were observed between the sediments, suggesting that this parameter likely did not play a significant role in influencing the outcomes of the toxicity assessments.

**Table 1:** Grain size distribution, organic matter (OM), calcium carbonate (CaCO₃) content, and wet weight percentages of the sediments collected in Cananéia (São Paulo, Brazil) and used in the dichlofluanid exposure assays with *Anomalocardia flexuosa*

	Gravel	Very Coarse	Coarse	Medium Sand	Fine Sand	Very Fine Sand	Silt & Clay	OM	CaCO_3_	Wet Weight
**High OM**	0.37	0.33	2.11	5.21	18.22	55.63	17.10	10.38	7.38	57.18
**Low OM**	0.00	0.07	0.62	1.42	8.66	74.53	13.81	4.00	5.88	49.52

OM: Organic Matter; CaCO_3_: Calcium carbonate

#### Chemical analyses

As previously reported by Campos et al. (2024) and summarized in Table 2, dichlofluanid was quantifiable only at a concentration of 1000 ng/g, where it exhibited degradation rates of 87% within 6 h and 99% within 24 h. This compound is recognized as a rapidly degrading biocide with a very short environmental half-life (Hamwijk et al. 2005). This rapid degradation, even at the 1000 ng/g, may represent a relevant limitation of studying dichlofluanid, as it suggests that organisms exposed to lower nominal concentrations experienced transient or negligible exposure to the parent compound, with potential implications for interpreting dose-response relationships. Nevertheless, given that dichlofluanid has been detected at concentrations exceeding established toxicity thresholds and its degradation can lead to the formation of potentially toxic transformation products, investigating its effects remains ecologically relevant.

**Table 2:** Measured concentrations of dichlofluanid at 0, 6, and 24 h post-spiking under equilibrium conditions, as previously reported by Campos et al. (2024)

	Nominal concentration (ng.g^− 1^)	Time		
		0 h	6 h	24 h
Dichlofluanid (DL = 0.5; QL = 1)	Blank (0)	< QL	< DL	< QL
	10	< DL	< QL	< DL
	100	< QL	< QL	< QL
	1000	1189.5 ± 460.4	157.7 ± 36	16.9 ±7.4

DL= Detection limit; QL – Quantification limit

#### Exposure of *Anomalocardia flexuosa*

No mortality was observed among organisms exposed to sediments enriched with high OM content. In contrast, clams exposed to low-OM sediment exhibited some mortality; however, the rates remained below 30% (Figure S2). The physicochemical parameters of the overlying water within the test-chambers during the test are presented in Table S5 and remained within suitable ranges for the species (Cruz et al. 2021).

### Biomarkers of exposure and effect – *Anomalocardia flexuosa*

**Fig. 5:**
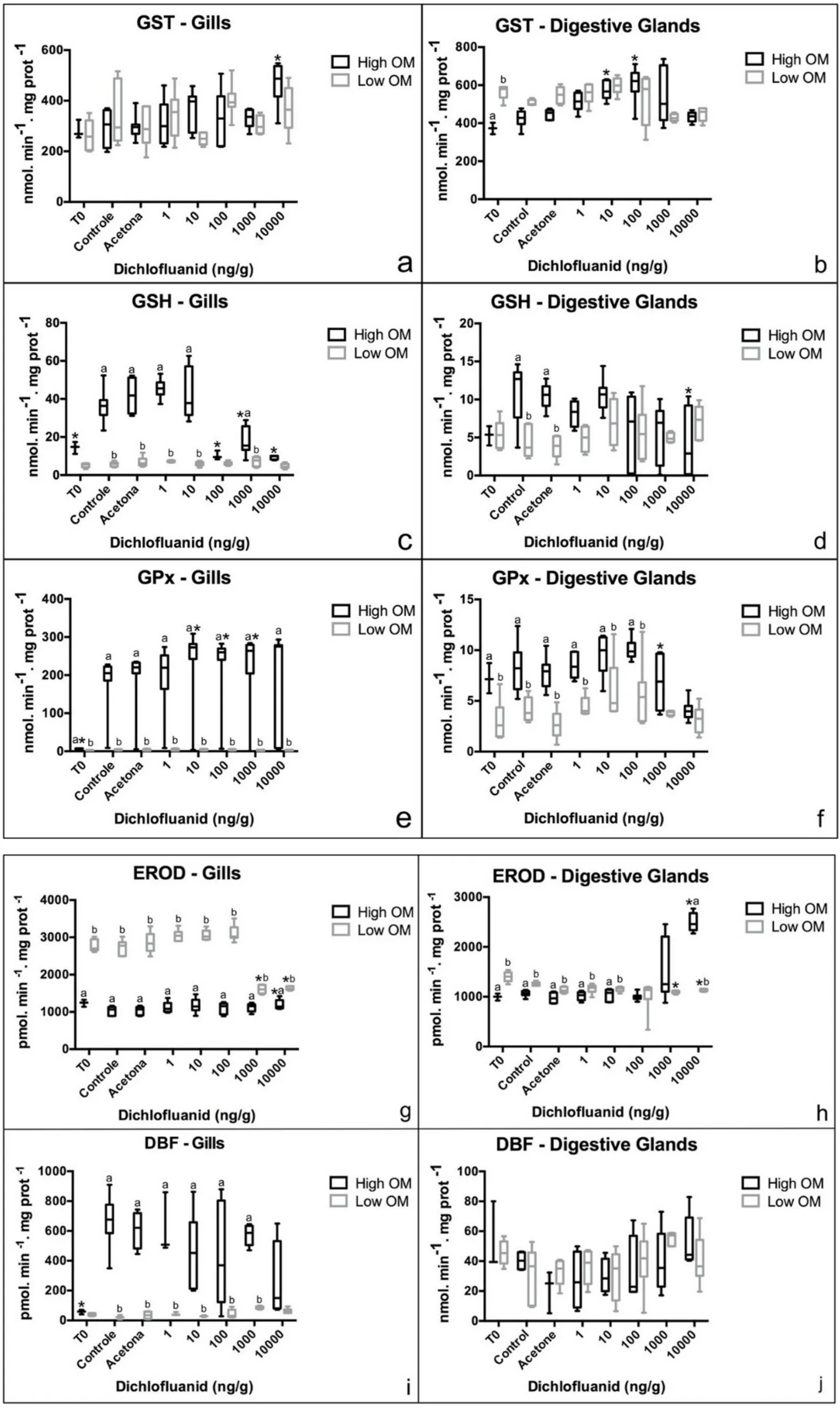
Biomarker responses of exposure in gills and digestive glands of the clam *Anomalocardia flexuosa* exposed to dichlofluanid through spiked sediments (*n* = 42 per treatment). Data are presented as box plots, with boundaries indicating the 25th and 75th percentiles; the line within each box marks the median value. An asterisk (*) above the data points indicates a statistically significant difference compared to the acetone control within a specific organic matter condition. Letters denote significant differences between organic matter levels at the same dichlofluanid concentration (*p* = 0.05). High OM = High organic matter; Low OM = Low organic matter. Figure 5.a. ANOVA, *p* = 0.0035; Fig. 5.b. ANOVA, *p* < 0.0001; Fig. 5.c. ANOVA, *p* < 0.0001; Fig. 5.d. ANOVA, *p* = 0.0057; Fig. 5.e. PERMANOVA, *p* = 0.001; Fig. 5.f. ANOVA, *p* < 0.0001; Fig. 5.g. PERMANOVA, *p* = 0.001; Fig. 5.h. PERMANOVA, *p* = 0.001; Fig. 5.i. ANOVA, *p* = 0.0089; Fig. 5.j. ANOVA, *p* = 0.3946

**Fig. 6:**
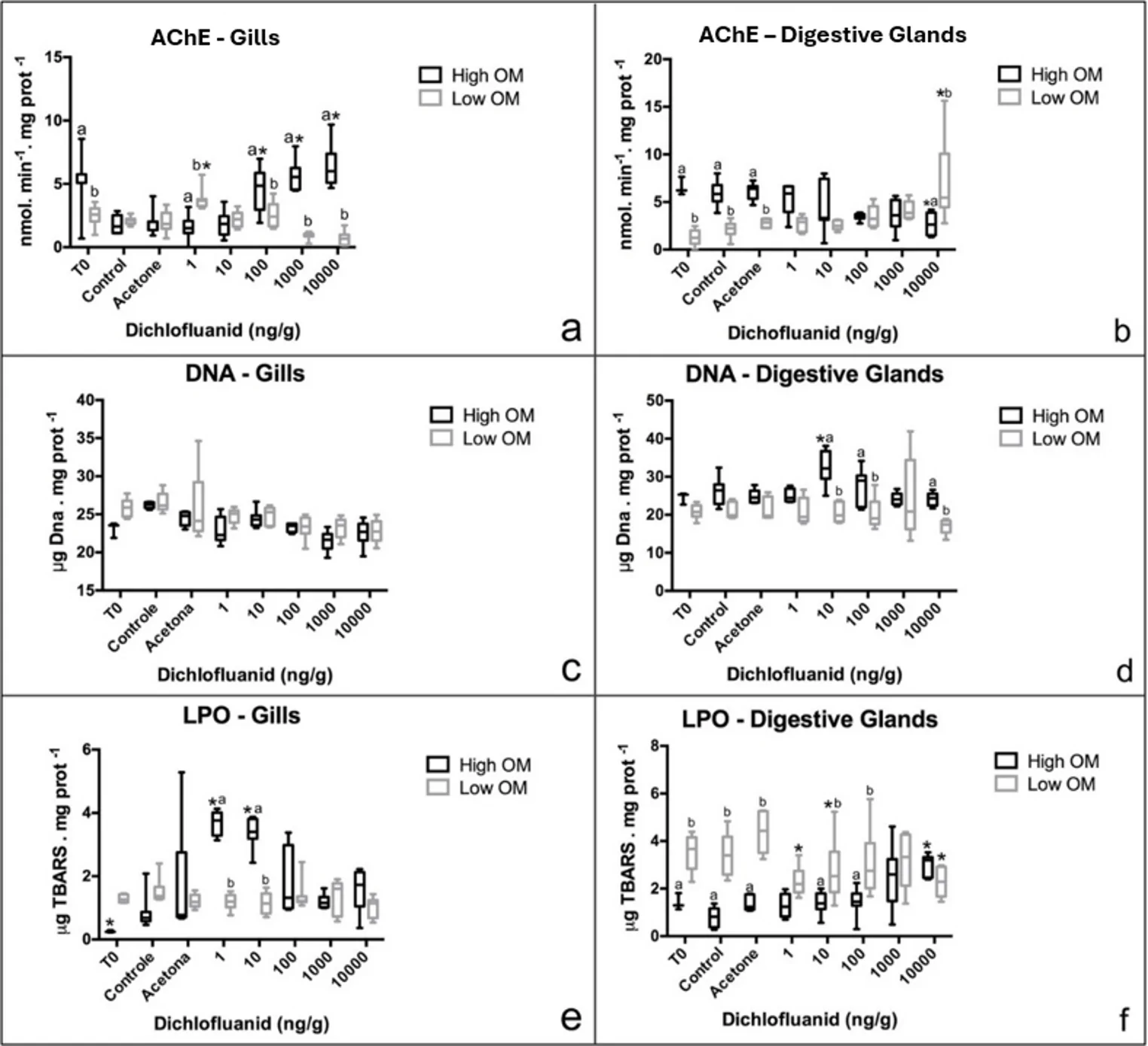
Biomarker responses of effect in gills and digestive glands of the clam *Anomalocardia flexuosa* exposed to dichlofluanid through spiked sediments (*n* = 42 per treatment). Data are presented as box plots, with boundaries indicating the 25th and 75th percentiles; the line within each box represents the median value. An asterisk (*) above the data indicates statistically significant differences compared to the acetone control within a given level of organic matter. Letters denote statistically significant differences between organic matter levels for the same dichlofluanid concentration (*p* = 0.05). High OM = High organic matter; Low OM = Low organic matter. Two-way ANOVA: Fig. 6.a. *p* < 0.0001; Fig. 6.b. *p* < 0.0001; Fig. 6.c. *p* = 0.5284; Fig. 6.d. *p* = 0.0170; Fig. 6.e. *p* < 0.0001; Fig. 6.f. *p* < 0.0001

Figures 5 and 6 show the biomarker responses measured in *A. flexuosa* following exposure to dichlofluanid-spiked sediments. In gill tissues, sediments with high OM content induced an increase in GST and EROD activities at the highest concentration (10000 ng/g), a reduction in GSH levels, and elevated GPx and AChE activities at the three highest concentrations (100, 1000, and 10000 ng/g). Increased LPO levels were also observed at intermediate concentrations (1 and 10 ng/g), at high OM sediments. In the organisms exposed to sediments with low OM content, no significant differences were observed in most biomarkers (GST and GPx activities, GSH levels, EROD, DBF, AChE activity, and DNA damage). Exposure to low-OM sediment led to a reduction in EROD activity at the highest concentrations (1000 and 10000 ng/g).

In the digestive glands, exposure to high-OM sediment led to enhanced GST activity at intermediate concentrations (10 and 100 ng/g), decreased GSH and AChE activity, increased EROD activity, and LPO levels at 10,000 ng/g, decreased GPx activity at 1000 ng/g, and increased DNA damage at 10 ng/g. No differences were observed for DBF, in both types of sediments. For low-OM sediment, the results showed decreased EROD activity at 1000 and 10,000 ng/g, increased AChE activity at 10,000 ng/g, and reduced LPO levels at varying concentrations (1, 10, and 10000 ng/g) of Dichlofluanid. Other biomarkers (GST, GPx, GSH, DNA damage) did not exhibit significant differences compared to the controls.

Overall, both the gills and digestive glands exhibited consistent evidence of the activation of defense mechanisms, such as alteration of biotransformation processes (EROD), stimulation of conjugation mechanisms (GST activity), alteration of antioxidant responses (GPx, GSH), associated cellular effects (DNA damage and LPO), and altered AChE activity, which may indicate potential neurotoxicity. These effects tended to be more pronounced in animals exposed to higher concentrations of dichlofluanid and organically richer sediments (i.e., high OM), contradicting our initial hypothesis.

### Principal component analysis (PCA) – *Anomalocardia flexuosa*

**Fig. 7:**
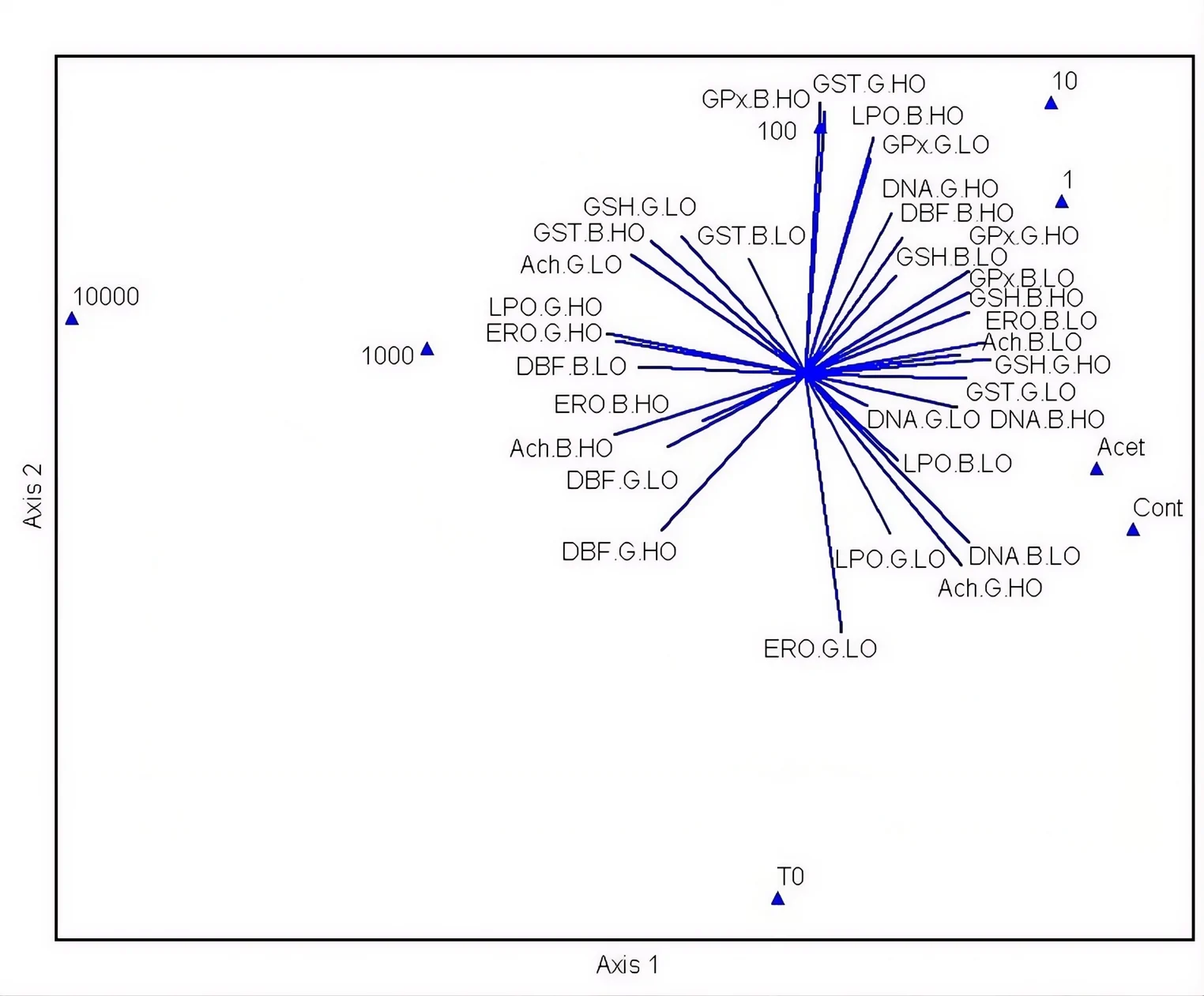
Projection of variables on a plane formed by factors 1 and 2 resulting of Principal Components Analysis (PCA) integrating all biomarkers analyzed in *Anomalocardia flexuosa* exposed to various dichlofluanid concentrations in spiked sediments. G = Gills; DG = Digestive Gland; H = High organic matter; L = Low organic matter

The results of the PCA performed using the results obtained from the exposure tests with sediments are presented in Tables S6 and S7 and Fig. 7. The first two principal components accounted for 63.93% of the total variance (Table S6). PCA revealed that along PC1, the water control, acetone blank, T0, and lower dichlofluanid concentrations (i.e., 1 and 10 ng/g) clustered together, whereas the higher concentrations (100, 1000, and 10000 ng/g) formed separate groups (Table S6, Fig. 7). Along PC2, T0, water control, and the acetone blank were grouped together and separated from the 1 and 10 ng/g concentrations, whereas the remaining dichlofluanid concentrations formed a distinct cluster (Table S6).

For PC1, in sediments with high organic matter content, the gill tissues showed a positive association between GST and AChE and a negative association between GSH and DNA damage. In the digestive glands, EROD and lipid peroxidation (LPO) were positively associated, whereas glutathione (GSH), glutathione peroxidase (GPx), and acetylcholinesterase (AChE) showed inverse associations. In sediments with low organic matter, gills exhibited associations among EROD, GPx, DNA damage, AChE, and LPO; however, most of these variables did not show significant variations according to ANOVA (Figs. 5 and 6). An inverse relationship was observed between GST, GSH, and AChE in the digestive glands (Table S6). For PC2, in high organic matter sediments, gills showed an association between GPx and LPO, while in the digestive glands, GST and DNA damage were positively associated, and AChE was negatively correlated with DNA damage.

In the projection of the variables along the two principal components extracted by PCA, the data were grouped into three clusters plotted on the plane defined by PCs 1 and 2 (Fig. 7), similar to the pattern observed in the water exposure experiments. The T0 treatment appeared to be isolated from the other two treatments. The second cluster comprised the control, analytical blank, and lower dichlofluanid concentrations (1, 10, and 100 ng/g). The third group had the highest concentrations (1000 and 10000 ng/g) of the target compounds. These results also suggest a more pronounced metabolic effect in organisms exposed to higher dichlofluanid concentrations. Overall, the PCA results supported the occurrence of adverse effects in *A. flexuosa* individuals exposed to dichlofluanid through sediments, particularly under conditions of elevated levels of organic matter. These effects were more consistent at higher concentrations; however, biochemical alterations were detectable even at the lowest tested concentrations, indicating that the animals responded to minimal levels of dichlofluanid.

The results of PERMANOVA using data obtained for both gills and digestive glands are presented in the Supplementary Material (Figs S3 and S4, and Tabs S8-S11). In summary, these results showed a clear separation between the different types of sediments, with a grouping of high-OM sediment with the highest concentrations of low-OM sediment, whereas the remaining concentrations of low-OM sediment formed a second group for the gills. These results also highlight that for both high- and low-organic matter sediments, significant differences in biomarkers were observed at the highest concentrations. Similar results were observed for the digestive glands, indicating separation by sediment type (i.e., OM content), and only the highest dichlofluanid concentrations in the high-OM sediment were isolated. When comparing sediments with different organic matter concentrations, this analysis revealed significant differences in the controls and at concentrations of 1, 10, and 10,000 ng/g.

In general, biological responses were more pronounced in organisms exposed to sediments with high quantities of organic matter. In such matrices, organic compounds tend to adsorb onto organic matter, establishing various chemical interactions that directly influence their bioavailability and toxicity to benthic organisms (Haitzer et al. 1998; Jones and De Voogt 1999). The stability of contaminants in sedimentary matrices is also related to the rate and degree of organic matter decomposition (Said-Pullicino et al. 2007). This fraction, which is retained in the sediment, can be directly transferred to benthic organisms, such as *A. flexuosa* in the present study. According to Lagreze et al. (2022), *A. flexuosa* is a filter-feeding mollusk that ingests particles suspended in water using its siphons; however, it also ingests large amounts of organic and inorganic material deposited on the sediments (Rodrigues et al. 2010) and is thus susceptible to contaminants present in the sedimentary environment.

Other booster biocides used in antifouling paints with solubilities comparable to that of dichlofluanid, such as diuron (log Kow = 2.7–2.8) and TBT (log Kow = 3.1–4.11), also appear to be strongly influenced by the concentration of organic matter, with higher OM levels enhancing their retention (Rocha et al. 2013; Furdek et al. 2016). In this sense, the increased retention of dichlofluanid in OM richer sediments likely favors the exposure of *A. flexuosa* and consequently could explain the negative effects recorded in our study, especially at higher concentrations.

Biomarker analyses revealed a typical response pattern in both the gills and digestive glands: activation of EROD, which is involved in phase I biotransformation processes, followed by the induction of conjugation enzymes such as GST, accompanied by the consumption of GSH during detoxification. However, the antioxidant system was insufficient to completely metabolize or eliminate dichlofluanid, resulting in increased lipid peroxidation. This outcome is consistent with the findings of Suzuki et al. (2004) in rat hepatocytes, who demonstrated that dichlofluanid induced lipid peroxidation and cytotoxicity at most of the tested concentrations above 25 µM. In addition to oxidative stress, signs of neurotoxicity were observed in both the tissues. A key target of some pesticides, particularly organophosphates, is the enzyme acetylcholinesterase (AChE), which hydrolyzes the neurotransmitter acetylcholine, and increased AChE activity has been associated with cellular apoptosis (Zhang et al. 2002). In this study, an increase in AChE activity was reported in both the gills and digestive glands, associated with biomarkers related to oxidative stress, such as LPO and DNA damage. This can be explained by the mode of action of dichlofluanid, which involves increased production of ROS, causing oxidative stress (Keppler and Ringwood 2001; Lushchak et al. 2018). Therefore, our results demonstrate that dichlofluanid can harm neotropical benthic species such as *A. flexuosa*, especially at higher concentrations and in organically rich sediments. However, further studies using other taxonomic groups are necessary to detail the ecological risks caused by dichlofluanid to tropical species.

## Conclusion

Based on these results, it can be inferred that dichlofluanid induces cellular-level effects in both target and nontarget species; i.e., organisms that form biofouling (mussels) and those that do not (clams), respectively in this study. In mussels, effects were observed from 0.1 µg/L (against a maximum measured environmental concentration of 3.37 µg/L (Campos et al. 2022a), whereas in clams, effects were observed from 1 µg/g. While this concentration is slightly above the maximum measured environmental concentration (MEC) for dichlofluanid in sediments (0.8 µg/g), it indicates potential risk in scenarios of localized higher contamination or underestimation of MECs. These effects were more pronounced and evident in sediment exposures, which may be attributed to the tendency of the compounds to adsorb onto sediment particles. Furthermore, organisms exposed to sediments with high organic matter content exhibited more intense alterations than those exposed to sediments with low organic matter content. Considering the full set of biomarker responses, the importance of using an integrated biomarker approach is evident, as it enables early warning signals for aquatic environmental monitoring. Finally, this study contributes new, crucial data to the understanding of dichlofluanid ecotoxicity in tropical marine environments, particularly given the previous knowledge gap. Because such effects were observed at environmental concentrations, ecological risks are likely to occur; therefore, actions are needed to regulate this substance.

## Supplementary information

Below is the link to the electronic supplementary material.

Supplementary Material 1

## Data Availability

All data generated or analyzed during this study are included in this published article and its supplementary information files.
