# Supplementary material for: Effects of dichlofluanid in tropical marine bivalves exposed to water and spiked sediments: an assessment of biomarker responses
Source: Ecotoxicology. 2026 Oct 1;35(8):180. doi: 10.1007/s10646-026-03164-y (PMC13630793; doi:10.1007/s10646-026-03164-y)
Supplement: Supplementary file 1 [file 10646_2026_3164_MOESM1_ESM.docx]

**Effects of Dichlofluanid in tropical marine bivalves exposed to water and spiked sediments: an assessment of biomarker responses**

Cruz, A.C.F.; Gusso-Choueri, P.K.; Pauly, G.F.E.; Campos, B.G.; Perina, F.C.; Castro, Í.B.; Abessa, D.M.S.

Supplementary Material

Table S1. Physicochemical parameters of the seawater in the mussel farm of *Perna perna* in Cocanha Beach, Caraguatatuba, north shore of São Paulo, Brazil.

| **Physicochemical parameters – Cocanha Beach** | |
| --- | --- |
| **pH** | 8.9 |
| **Salinity** | 33 |
| **Dissolved Oxygen (mg/L)** | 5.6 |

Table S2. Physicochemical parameters of test-solution during the experiments conducted with *Perna perna.* Nominal Dichlofluanid concentrations are expressed as µg/L. D.O. = dissolved oxygen.

| **Day 1** | | | | | | |
| --- | --- | --- | --- | --- | --- | --- |
| **Conc. (µg/L)** | **Salinity** | **pH** | **D.O. (mg/L)** | **Temperature (°C)** | **Total ammonia (mg/L)** | **Unionized ammonia (µg/L)** |
| Control | 33 | 7.2 | 3.8 | 25±2 | 0.00100 | 0.007 |
| Acetone | 34 | 7.3 | 3.7 | 25±2 | 0.00118 | 0.011 |
| 0.01 | 35 | 7.4 | 3.5 | 25±2 | 0.00103 | 0.012 |
| 0.1 | 35 | 7.6 | 3.5 | 25±2 | 0.00100 | 0.018 |
| 1 | 35 | 7.5 | 3.3 | 25±2 | 0.00058 | 0.008 |
| 10 | 35 | 7.5 | 3.1 | 25±2 | 0.00094 | 0.013 |
| 100 | 35 | 7.5 | 3.4 | 25±2 | 0.00052 | 0.007 |
| **Day 2** | | | | | | |
| **Conc. (µg/L)** | **Salinity** | **pH** | **D.O. (mg/L)** | **Temperature (°C)** | **Total ammonia (mg/L)** | **Unionized ammonia (µg/L)** |
| Control | 34 | 7.67 | 4.4 | 25±2 | 0.0026 | 0.330 |
| Acetone | 33 | 7.67 | 2.3 | 25±2 | 0.0007 | 0.079 |
| 0.01 | 33 | 7.86 | 3.7 | 25±2 | 0.0006 | 0.012 |
| 0.1 | 33 | 7.8 | 2.5 | 25±2 | 0.0006 | 0.013 |
| 1 | 33 | 7.86 | 3.4 | 25±2 | 0.0006 | 0.019 |
| 10 | 33 | 8.03 | 2.2 | 25±2 | 0.0005 | 0.013 |
| 100 | 33 | 8.04 | 2.9 | 25±2 | 0.0004 | 0.020 |
| **Day 3** | | | | | | |
| **Conc. (µg/L)** | **Salinity** | **pH** | **D.O. (mg/L)** | **Temperature (°C)** | **Total ammonia (mg/L)** | **Unionized ammonia (µg/L)** |
| Control | 33 | 8.21 | 4.2 | 25±2 | 0.00114 | 0.055 |
| Aceton | 33 | 8.51 | 4.7 | 25±2 | 0.00023 | 0.016 |
| 0.01 | 33 | 8.62 | 4.3 | 25±2 | 0.00033 | 0.054 |
| 0.1 | 33 | 8.66 | 4.6 | 25±2 | 0.00023 | 0.040 |
| 1 | 34 | 8.67 | 4.6 | 25±2 | 0.00018 | 0.032 |
| 10 | 34 | 7.59 | 4.5 | 25±2 | 0.00024 | 0.004 |
| 100 | 34 | 7.56 | 4.9 | 25±2 | 0.00025 | 0.004 |
| **Day 4** | | | | | | |
| **Conc. (µg/L)** | **Salinity** | **pH** | **D.O. (mg/L)** | **Temperature (°C)** | **Total ammonia (mg/L)** | **Unionized ammonia (µg/L)** |
| Control | 34 | 7.25 | 4.6 | 25±2 | 0.00114 | 0.055 |
| Acetone | 33 | 7.32 | 4.5 | 25±2 | 0.00023 | 0.016 |
| 0.01 | 33 | 7.02 | 4 | 25±2 | 0.00033 | 0.018 |
| 0.1 | 33 | 7.26 | 4.4 | 25±2 | 0.00039 | 0.017 |
| 1 | 33 | 7.80 | 4.1 | 25±2 | 0.00035 | 0.019 |
| 10 | 33 | 7.04 | 4.5 | 25±2 | 0.00011 | 0.022 |
| 100 | 34 | 7.14 | 4.4 | 25±2 | 0.00015 | 0.020 |

Figure S1. Mortality rates of *Perna perna* mussels during the 96-h experiment of Dichlofluanid exposure.

Table S3. Results of Principal Components Analysis (PCA) integrating all biomarkers analyzed in *Perna perna* mussels exposed to dichlofluanid concentrations in water. Factorial Scores for the initial time, control, acetone control, and dichlofluanid concentrations at 96 h in the water exposures. G = Gills; DG = Digestive Gland; NR = Neutral Red. Bold values indicate significant correlations with the respective PCs.

| PC | Eigenvalue | | % Variance | | Cumulative Variance |
| --- | --- | --- | --- | --- | --- |
| PC1 | 6.12 | | 35.97 | | 35.97 |
| PC2 | 4.86 | | 28.58 | | 64.55 |
| Variable | | PC1 | | PC2 | |
| DNA.G | | -0.24 | | **-0.75** | |
| LPO. G | | -0.49 | | 0.30 | |
| AchE. G | | 0.29 | | **0.90** | |
| Erod. G | | **-0.61** | | 0.38 | |
| DBF G | | 0.10 | | 0.27 | |
| GPx. G | | **-0.87** | | 0.15 | |
| GSH. G | | **-0.83** | | 0.22 | |
| GST. G | | -0.42 | | **-0.65** | |
| DNA.DG | | -0.46 | | **0.69** | |
| LPO.DG | | 0.31 | | **0.83** | |
| AchE.DG | | 0.46 | | **0.56** | |
| Erod.DG | | 0.12 | | **0.63** | |
| DBF DG | | **0.87** | | -0.36 | |
| GPx.DG | | **-0.53** | | **0.56** | |
| GSH.DG | | **0.91** | | -0.21 | |
| GST.DG | | **-0.93** | | -0.11 | |
| NRRT | | **0.69** | | **0.55** | |
| Treatment | | PC1 | | PC2 | |
| T0 | | 2.31 | | -4.80 | |
| T96_Control | | 1.70 | | 0.53 | |
| T96_Acetone | | 2.67 | | 2.98 | |
| T96_0.01 | | 2.38 | | 1.01 | |
| T96_0.1 | | -1.56 | | 0.78 | |
| T96_1 | | -1.90 | | -0.43 | |
| T96_10 | | -2.72 | | -0.02 | |
| T96_100 | | -2.88 | | -0.05 | |

Table S4. Additional products from the PCA performed with the results of the experiment exposing adults of the mussel *Perna perna* to dichlofluanid through water.

| **Axis** | **Eigenvalue** | **Eigenvalues from randomizations** | | | **p *** |
| --- | --- | --- | --- | --- | --- |
|  |  | **Minimum** | **Average** | **Maximum** |  |
| 1 | 6.1146 | 3.6041 | 4.8870 | 7.4203 | 0.012 |
| 2 | 4.8584 | 2.7826 | 3.7270 | 4.8869 | 0.002 |
| 3 | 2.4492 | 1.7284 | 2.8882 | 3.8736 | 0.934 |
| 4 | 1.5401 | 1.3494 | 2.2013 | 2.9921 | 0.993 |
| 5 | 1.2412 | 0.81917 | 1.5976 | 2.4164 | 0.923 |
| 6 | 0.43427 | 0.47288 | 1.0872 | 1.9531 | 1.000 |
| 7 | 0.36221 | 0.15394 | 0.61181 | 1.2868 | 0.882 |
| 8 | 0.10754E-11 | 0.23194E-12 | 0.82376E-12 | 0.13197E-11 | 0.109 |
| 9 | 0.19001E-15 | 0.10727E-15 | 0.27982E-15 | 0.58006E-15 | 0.924 |
| 10 | 0.13820E-15 | 0.62384E-16 | 0.18188E-15 | 0.35489E-15 | 0.796 |
| * p-value for an axis is (n+1)/(N+1), where n is the number of randomizations with an eigenvalue for that axis that is equal to or larger than the observed eigenvalue for that axis. N is the total number of randomizations. | | | | | |

**Figure S2**. Mortality rates of *Anomalocardia flexuosa* clams along the 21-day experiment exposing the organisms using the sediment with low organic matter quantities. No mortalities were observed among clams exposed to the organically richer sediments.

Table S5. Physicochemical parameters of the overlying water within the test-chambers during the experiments conducted with *Anomalocardia flexuosa.* Measurements were made at the start of the experiment (T0) and after 7, 14, and 21 days. Nominal Dichlofluanid concentrations are expressed as ng/g. D.O. = dissolved oxygen.

| **Sediment with high organic matter level (High OM)** | | | | | | |
| --- | --- | --- | --- | --- | --- | --- |
| T0 | | | | | | |
| **Conc (ng/g)** | **pH** | **Salinity** | **D.O. (mg/L)** | **Temperature (°C)** | **Total ammonia (mg/L)** | **Ionized ammonia (µg/L)** |
| Control | 7.65±0.03 | 29.33±0.52 | 6.82±0.53 | 25±2 | 0.00054±0.00009 | 0.01113±0.0014 |
| Acetone | 7.61±0.09 | 30.00±0.00 | 6.20±0.62 | 25±2 | 0.00040±0.0001 | 0.00728±0.001 |
| 1 | 7.60±0.02 | 30.00±0.00 | 6.07±0.69 | 25±2 | 0.00041±0.0001 | 0.00739±0.0016 |
| 10 | 7.61±0.11 | 30.00±0.00 | 6.12±0.99 | 25±2 | 0.00036±0.00007 | 0.00698±0.0019 |
| 100 | 7.59±0.05 | 29.50±0.84 | 6.82±0.46 | 25±2 | 0.00044±0.00003 | 0.00805±0.0013 |
| 1000 | 7.61±0.06 | 29.67±0.52 | 6.63±0.29 | 25±2 | 0.00058±0.0001 | 0.01098±0.0026 |
| 10000 | 7.57±0.03 | 29.17±0.41 | 6.57±0.75 | 25±2 | 0.00047±0.00007 | 0.00822±0.0016 |
| T7 | | | | | | |
| **Conc (ng/g)** | **pH** | **Salinity** | **D.O. (mg/L)** | **Temperature (°C)** | **Total ammonia (mg/L)** | **Ionized ammonia (µg/L)** |
| Control | 7.58±0.11 | 30.1±2.13 | 6.67±0.46 | 25±2 | 0.0020±0.0009 | 0.0389±0.022 |
| Acetone | 7.63±0.11 | 30.1±1.23 | 6.36±0.65 | 25±2 | 0.0009±0.0004 | 0.0188±0.010 |
| 1 | 7.52±0.13 | 29.6±0.70 | 6.47±0.35 | 25±2 | 0.0017±0.0007 | 0.0264±0.012 |
| 10 | 7.61±0.08 | 29.2±1.14 | 6.5±0.69 | 25±2 | 0.0015±0.0007 | 0.0285±0.010 |
| 100 | 7.57±0.09 | 29.2±1.23 | 6.02±0.24 | 25±2 | 0.0009±0.0004 | 0.0155±0.005 |
| 1000 | 7.56±0.14 | 28.9±1.10 | 5.86±0.25 | 25±2 | 0.0020±0.0008 | 0.0336±0.015 |
| 10000 | 7.60±0.08 | 29.2±1.03 | 5.5±0.25 | 25±2 | 0.0011±0.0005 | 0.0206±0.014 |
| T14 | | | | | | |
| **Conc (ng/g)** | **pH** | **Salinity** | **D.O. (mg/L)** | **Temperature (°C)** | **Total ammonia (mg/L)** | **Ionized ammonia (µg/L)** |
| Control | 7.56±0.06 | 33.00±0.63 | 5.52±1.09 | 25±2 | 0.0006±0.0003 | 0.009±0.004 |
| Acetone | 7.62±0.03 | 30.33±0.82 | 5.30±1.00 | 25±2 | 0.0013±0.0005 | 0.025±0.008 |
| 1 | 7.60±0.10 | 30.83±1.17 | 5.37±1.10 | 25±2 | 0.0008±0.0003 | 0.014±0.004 |
| 10 | 7.60±0.02 | 30.39±1.21 | 5.21±0.97 | 25±2 | 0.0010±0.0003 | 0.020±0.005 |
| 100 | 7.70±0.11 | 30.33±1.03 | 5.57±0.84 | 25±2 | 0.0010±0.0006 | 0.023±0.014 |
| 1000 | 7.64±0.18 | 30.33±0.82 | 5.68±0.99 | 25±2 | 0.0007±0.0007 | 0.010±0.008 |
| 10000 | 7.65±0.14 | 30.00±0.00 | 5.52±0.87 | 25±2 | 0.0014±0.0004 | 0.028±0.006 |
| T21 | | | | | | |
| **Conc (ng/g)** | **pH** | **Salinity** | **D.O. (mg/L)** | **Temperature (°C)** | **Total ammonia (mg/L)** | **Ionized ammonia (µg/L)** |
| Control | 7.48±0.12 | 33.50±0.71 | 5.90±0.57 | 25±2 | 0.0003±0.0003 | 0.0045±0.005 |
| Acetone | 7.56±0.30 | 32.00±0.00 | 4.85±0.35 | 25±2 | 0.0007±0.0003 | 0.0149±0.014 |
| 1 | 7.60±0.26 | 30.50±0.71 | 5.00±0.14 | 25±2 | 0.0003±0.0001 | 0.0056±0.001 |
| 10 | 7.56±0.22 | 31.00±0.00 | 4.80±0.57 | 25±2 | 0.0004±0.00001 | 0.0067±0.003 |
| 100 | 7.74±0.05 | 31.00±0.00 | 5.10±0.57 | 25±2 | 0.0004±0.00005 | 0.0087±0.0004 |
| 1000 | 7.76±0.07 | 30.50±0.71 | 5.50±0.42 | 25±2 | 0.0002±0.00005 | 0.0045±0.0006 |
| 10000 | 7.84±0.06 | 31.00±0.00 | 5.25±0.07 | 25±2 | 0.0007±0.0004 | 0.0212±0.014 |
| **Sediment with low organic matter quantity (Low OM)** | | | | | | |
| T0 | | | | | | |
| **Conc (ng/g)** | **pH** | **Salinity** | **D.O. (mg/L)** | **Temperature (°C)** | **Total ammonia (mg/L)** | **Ionized ammonia (µg/L)** |
| Control | 7.69±0.12 | 30.50±0.55 | 4.42±0.39 | 25±2 | 0.0021±0.0014 | 0.055±0.04 |
| Acetone | 7.78±0.05 | 30.50±0.55 | 5.90±0.26 | 25±2 | 0.0018±0.0002 | 0.050±0.01 |
| 1 | 7.79±0.04 | 31.17±0.41 | 6.22±0.16 | 25±2 | 0.0015±0.0002 | 0.042±0.01 |
| 10 | 7.78±0.07 | 31.83±0.41 | 5.83±0.45 | 25±2 | 0.0012±0.0004 | 0.035±0.01 |
| 100 | 7.77±0.09 | 31.17±0.75 | 5.82±0.48 | 25±2 | 0.0010±0.0004 | 0.029±0.01 |
| 1000 | 7.77±0.05 | 30.33±0.52 | 5.45±0.45 | 25±2 | 0.0008±0.0003 | 0.022±0.01 |
| 10000 | 7.71±0.05 | 30.33±0.52 | 5.17±1.12 | 25±2 | 0.0006±0.0002 | 0.013±0.003 |
| T7 | | | | | | |
| **Conc (ng/g)** | **pH** | **Salinity** | **D.O. (mg/L)** | **Temperature (°C)** | **Total ammonia (mg/L)** | **Ionized ammonia (µg/L)** |
| Control | 8.04±0.07 | 31.50±0.84 | 4.75±0.34 | 25±2 | 0.0020±0.002 | 0.11±0.095 |
| Acetone | 8.04±0.16 | 31.17±0.41 | 4.05±0.68 | 25±2 | 0.0020±0.0004 | 0.11±0.021 |
| 1 | 8.16±0.10 | 32.33±0.52 | 5.08±0.78 | 25±2 | 0.0023±0.0007 | 0.12±0.046 |
| 10 | 8.15±0.11 | 32.17±0.41 | 5.43±0.32 | 25±2 | 0.0025±0.0013 | 0.14±0.078 |
| 100 | 8.10±0.09 | 32.33±0.52 | 5.37±0.51 | 25±2 | 0.0022±0.0009 | 0.13±0.052 |
| 1000 | 8.12±0.13 | 32.00±0.89 | 6.37±0.16 | 25±2 | 0.0020±0.0004 | 0.11±0.019 |
| 10000 | 7.92±0.18 | 31.17±0.98 | 4.58±0.56 | 25±2 | 0.0020±0.0005 | 0.10±0.025 |
| T14 | | | | | | |
| **Conc (ng/g)** | **pH** | **Salinity** | **D.O. (mg/L)** | **Temperature (°C)** | **Total ammonia (mg/L)** | **Ionized ammonia (µg/L)** |
| Control | 7.95±0.27 | 32.17±1.72 | 5.23±0.47 | 25±2 | 0.0002 | 0.01 |
| Acetone | 7.97±0.28 | 31.50±1.87 | 4.92±0.80 | 25±2 | 0.0042±0.002 | 0.11±0.04 |
| 1 | 8.04±0.27 | 32.50±1.22 | 5.33±0.97 | 25±2 | <LD | <LD |
| 10 | 7.83±0.25 | 32.67±1.03 | 5.44±0.44 | 25±2 | 0.0033 | 0.09 |
| 100 | 7.96±0.30 | 33.33±1.03 | 5.77±0.23 | 25±2 | 0.0005±0.0005 | 0.01±0.01 |
| 1000 | 8.01±0.27 | 32.00±0.89 | 5.43±0.34 | 25±2 | 0.0013 | 0.02 |
| 10000 | 7.85±0.35 | 32.17±0.75 | 5.30±0.33 | 25±2 | 0.0013±0.0009 | 0.02±0.01 |
| T21 | | | | | | |
| **Conc (ng/g)** | **pH** | **Salinity** | **D.O. (mg/L)** | **Temperature (°C)** | **Total ammonia (mg/L)** | **Ionized ammonia (µg/L)** |
| Control | 8.29±0.18 | 33.50±0.71 | 5.40±0.14 | 25±2 | <LD | <LD |
| Aceton | 8.31±0.06 | 33.50±0.71 | 5.65±0.92 | 25±2 | <LD | <LD |
| 1 | 8.38±0.00 | 33.00±0.00 | 6.55±0.07 | 25±2 | <LD | <LD |
| 10 | 8.31±0.03 | 34.00±0.00 | 6.15±0.07 | 25±2 | <LD | <LD |
| 100 | 8.34±0.06 | 34.00±0.00 | 6.00±0.14 | 25±2 | <LD | <LD |
| 1000 | 8.35±0.04 | 32.00±0.00 | 5.80±0.28 | 25±2 | <LD | <LD |
| 10000 | 8.28±0.11 | 32.50±0.71 | 5.50±0.00 | 25±2 | <LD | <LD |

Table S6. Results of Principal Components Analysis (PCA) integrating all biomarkers analyzed in *Anomalocardia flexuosa* individuals exposed to dichlofluanid concentrations in spiked sediments with two matrices of differing organic matter content. Factor scores for the initial time point, control, acetone blank, and dichlofluanid concentrations in sediment exposures over 21 days (Concentrations in ng/g). G = Gills; DG = Digestive gland; H = High organic matter; L = Low organic matter. Bold values indicate significant correlations with the respective PCs.

| PC | Eigenvalue | | % Variance | | Cumulative Variance |
| --- | --- | --- | --- | --- | --- |
| PC1 | 12.03 | | 42.96 | | 42.96 |
| PC2 | 5.87 | | 20.97 | | 63.97 |
|  | | PC1 | | PC2 | |
| DNA.G.H | | **-0.70** | | 0.03 | |
| DNA.G.L | | **-0.83** | | 0.45 | |
| LPO. G.H | | -0.21 | | **-0.85** | |
| LPO. G.L | | **-0.51** | | 0.28 | |
| AchE. G.H | | **0.86** | | 0.30 | |
| AchE. G.L | | **-0.74** | | -0.21 | |
| EROD. G.H | | 0.45 | | 0.06 | |
| EROD. G.L | | **-0.85** | | -0.26 | |
| GPx. G.H | | 0.05 | | **-0.82** | |
| GPx. G.L | | **-0.73** | | -0.38 | |
| GSH. G.H | | -**0.73** | | -0.29 | |
| GSH. G.L | | -0.42 | | -0.28 | |
| GST. G.H | | **0.83** | | -0.38 | |
| GST. G.L | | 0.31 | | -0.28 | |
| DNA.DG.H | | -0.35 | | **-0.66** | |
| DNA.DG.L | | -0.36 | | 0.14 | |
| LPO. DG.H | | **0.97** | | 0.003 | |
| LPO. DG.L | | -0.46 | | **0.56** | |
| AchE. DG.H | | **-0.81** | | **0.51** | |
| AchE. DG.L | | **0.90** | | -0.25 | |
| EROD. DG.H | | **0.94** | | 0.02 | |
| EROD. DG.L | | -0.42 | | **0.69** | |
| GPx. DG.H | | **-0.77** | | -0.46 | |
| GPx. DG.L | | -0.25 | | -**0.81** | |
| GSH. DG.H | | **-0.85** | | -0.14 | |
| GSH. DG.L | | **0.65** | | -0.48 | |
| GST. DG.H | | -0.01 | | **-0.86** | |
| GST. DG.L | | **-0.76** | | -0.18 | |
| Treatment | | PC1 | | PC2 | |
| T0 | | -0.85 | | 4.24 | |
| T21_Control | | -3.14 | | 1.38 | |
| T21_Acetone | | -2.39 | | 1.29 | |
| T21_1 | | -2.12 | | -1.89 | |
| T21_10 | | -1.73 | | -3.28 | |
| T21_100 | | 0.03 | | -2.22 | |
| T21_1000 | | 3.08 | | 0.50 | |
| T21_10000 | | 7.13 | | -0.02 | |

Table S7. Additional products from the PCA performed with the results of the experiment exposing individuals of the clam *Anomalocardia flexuosa* to dichlofluanid through spiked sediments.

| **Axis** | **Eigenvalue** | **Eigenvalues from randomizations** | | | **p *** |
| --- | --- | --- | --- | --- | --- |
|  |  | **Minimum** | **Average** | **Maximum** |  |
| 1 | 13.567 | 6.1158 | 7.8505 | 10.222 | 0.001 |
| 2 | 6.4550 | 5.0654 | 6.3790 | 8.5782 | 0.428 |
| 3 | 4.5705 | 3.8662 | 5.2763 | 6.5825 | 0.966 |
| 4 | 2.7282 | 3.1197 | 4.3339 | 5.4014 | 1.000 |
| 5 | 2.0377 | 2.2409 | 3.5258 | 4.5497 | 1.000 |
| 6 | 1.6933 | 1.2998 | 2.7075 | 3.6831 | 0.997 |
| 7 | 0.94820 | 0.70957 | 1.9270 | 2.9583 | 0.995 |
| 8 | 0.79159E-12 | 0.41396E-12 | 0.86348E-12 | 0.10649E-11 | 0.780 |
| 9 | 0.58680E-15 | 0.33294E-15 | 0.49025E-15 | 0.74820E-15 | 0.103 |
| 10 | 0.51660E-15 | 0.24859E-15 | 0.38846E-15 | 0.60306E-15 | 0.005 |
| * p-value for an axis is (n+1)/(N+1), where n is the number of randomizations with an eigenvalue for that axis that is equal to or larger than the observed eigenvalue for that axis. N is the total number of randomizations. | | | | | |

**Table S8**. Results of PERMANOVA comparing sediment types (low and high OM). Analyses were based on Euclidean distances considering the biomarkers assessed in gill tissues.

|  | | | Df | SS | MS | | Pseudo-F | P(Perm) | | Perms. | |
| --- | --- | --- | --- | --- | --- | --- | --- | --- | --- | --- | --- |
| Concentration | | | 6 | 5.6961E6 | 9.4936E5 | | 26.371 | 0.001 | | 998 | |
| OM | | | 1 | 3.9486E7 | 3.9486E7 | | 1096.8 | 0.001 | | 998 | |
| Co x MO | | | 6 | 7.3653E6 | 1.2276E6 | | 34.098 | **0.001** | | 998 | |
| Residual | | | 60 | 2.16E6 | 36001 | |  |  | |  | |
| Total | | | 73 | 6.0548E7 |  | |  |  | |  | |
| Term 'CoxMO' for pairs of levels of factor 'Concentration'  Within level 'Rich' of factor 'OM' | | | | | | | | | | | |
| Groups | | | | t | P(Perm) | Perms | | | P(MC) | |  |
| Acetone x Control | | | | 0.53529 | 0.752 | 126 | | | 0.737 | |  |
| Acetone x 1 | | | | 1.4836 | 0.122 | 408 | | | 0.151 | |  |
| Acetone x 10 | | | | 1.2852 | 0.255 | 125 | | | 0.241 | |  |
| Acetone x 100 | | | | 0.41792 | 0.9 | 56 | | | 0.816 | |  |
| Acetone x 1000 | | | | 1.5299 | 0.139 | 409 | | | 0.15 | |  |
| Acetone x 10000 | | | | 2.4075 | 0.004 | 561 | | | **0.017** | |  |
| Term 'CoxOM' for pairs of levels of factor 'Concentration'  Within level 'Low' of factor 'OM' | | | | | | | | | | | |
| Groups | | | | t | P(Perm) | Perms | | | P(MC) | |  |
| Acetone x Control | | | | 0.91217 | 0.451 | 414 | | | 0.413 | |  |
| Acetone x 1 | | | | 1.2326 | 0.246 | 413 | | | 0.246 | |  |
| Acetone x 10 | | | | 1.3915 | 0.184 | 405 | | | 0.2 | |  |
| Acetone x 100 | | | | 2.0424 | 0.052 | 747 | | | 0.056 | |  |
| Acetone x 1000 | | | | 6.7453 | 0.014 | 84 | | | **0.001** | |  |
| Acetone x 10000 | | | | 8.8525 | 0.002 | 407 | | | **0.001** | |  |
| Term 'CoxOM' for pairs of levels of factor 'OM'  Factor 'Concentration' | | | | | | | | | | | |
| Samples | | Groups | | t | P(Perm) | Perms | | | P(MC) | |  |
| Control | High x Low | | | 14.562 | 0.006 | 401 | | | **0.001** | |  |
| Acetone | High x Low | | | 13.179 | 0.003 | 408 | | | **0.001** | |  |
| 1 | High x Low | | | 16.899 | 0.003 | 415 | | | **0.001** | |  |
| 10 | High x Low | | | 18.472 | 0.007 | 126 | | | **0.001** | |  |
| 100 | High x Low | | | 13.882 | 0.012 | 120 | | | **0.001** | |  |
| 1000 | High x Low | | | 6.5776 | 0.016 | 84 | | | **0.001** | |  |
| 10000 | High x Low | | | 4.8678 | 0.004 | 579 | | | **0.001** | |  |

**Table S9.** Additional results of PERMANOVA performed with the dataset obtained for gills of *Anomalocardia flexuosa* in the experiment with sediments.

| **Matrix: Sediment/ Tissue: Gills/ PERMANOVA/ Permutacional MANOVA** | | | | | | | | | | | | | |
| --- | --- | --- | --- | --- | --- | --- | --- | --- | --- | --- | --- | --- | --- |
| *Resemblance worksheet*  Name: Resemblance  Data type: Distance  Selection: All  Resemblance: D1 Euclidean distance | | | | | | | | | | | | | |
| Sums of squares type: Type III (partial)  Fixed effects sum to zero for mixed terms  Permutation method: Permutation of residuals under a reduced model  Number of permutations: 999 | | | | | | | | | | | | | |
| Factors | | | Abbrev | | | | Type | | | | Levels | | |
| Concentration | | | Co | | | | Fixed | | | | 7 | | |
| OM | | | MO | | | | Fixed | | | | 2 | | |
| *Details of the expected mean squares (EMS) for the model* | | | | | | | | | | | | | |
| Source | | | | | | | SEM | | | | | | |
| Co | | | | | | | 1*V(Res) + 10.086*S(Co) | | | | | | |
| MO | | | | | | | 1*V(Res) + 34.764*S(MO) | | | | | | |
| Co x MO | | | | | | | 1*V(Res) + 5.0429*S(CoxMO) | | | | | | |
| Res | | | | | | | 1*V(Res) | | | | | | |
| *Construction of Pseudo-F ratio(s) from mean squares* | | | | | | | | | | | | | |
| Source | | Numerator | | | | Denominator | | Num.df | | | | Den.df | |
| Co | | 1*Co | | | | 1*Res | | 6 | | | | 60 | |
| MO | | 1*MO | | | | 1*Res | | 1 | | | | 60 | |
| Co x MO | | 1*Co x MO | | | | 1*Res | | 6 | | | | 60 | |
| *Estimates of components of variation* | | | | | | | | | | | | | |
| Source | | | | Estimate | | | | | | Sq.root | | | |
| S(Co) | | | | 90559 | | | | | | 300.93 | | | |
| S(MO) | | | | 1.1348E6 | | | | | | 1065.3 | | | |
| S(CoxMO) | | | | 2.3628E5 | | | | | | 486.09 | | | |
| V(Res) | | | | 36001 | | | | | | 189.74 | | | |
| *PAIR-WISE TESTS*  Term 'CoxMO' for pairs of levels of factor 'Concentration'  Within level 'Rich' of factor 'OM' | | | | | | | | | | | | | |
| Groups | | | | Denominator | | | | | | Den.df | | | |
| Control x Acetone | | | | 1*Res | | | | | | 8 | | | |
| Acetone x 1 | | | | 1*Res | | | | | | 9 | | | |
| Acetone x 10 | | | | 1*Res | | | | | | 8 | | | |
| Acetone x 100 | | | | 1*Res | | | | | | 6 | | | |
| Acetone x 1000 | | | | 1*Res | | | | | | 9 | | | |
| Acetone x 10000 | | | | 1*Res | | | | | | 10 | | | |
| *Average Distance between/within groups*  Within level 'Rich' of factor 'OM' | | | | | | | | | | | | | |
|  | Control | | Acetone | | 0.01 | | 0.1 | | 1 | | 10 | | 100 |
| Control | 149.94 | |  | |  | |  | |  | |  | |  |
| Acetone | 144.37 | | 174.15 | |  | |  | |  | |  | |  |
| 1.0 | 191.37 | | 211.26 | | 228.5 | |  | |  | |  | |  |
| 10.0 | 198.95 | | 215.62 | | 214.62 | | 247.93 | |  | |  | |  |
| 100.0 | 193.67 | | 189.56 | | 247.11 | | 243.7 | | 251.44 | |  | |  |
| 1000.0 | 166.79 | | 184.06 | | 182.9 | | 187.73 | | 207.12 | | 159.47 | |  |
| 10000.0 | 273.99 | | 308.2 | | 266.44 | | 263.08 | | 333.56 | | 251.72 | | 251.78 |
| *PAIR-WISE TESTS*  Term 'CoxMO' for pairs of levels of factor 'Concentration'  Within level 'Poor' of factor 'OM' | | | | | | | | | | | | | |
| Groups | | | | Denominator | | | | | | Den.df | | | |
| Control x Acetone | | | | 1*Res | | | | | | 10 | | | |
| Acetone x 1 | | | | 1*Res | | | | | | 9 | | | |
| Acetone x 10 | | | | 1*Res | | | | | | 9 | | | |
| Acetone x 100 | | | | 1*Res | | | | | | 11 | | | |
| Acetone x 1000 | | | | 1*Res | | | | | | 7 | | | |
| Acetone x 10000 | | | | 1*Res | | | | | | 9 | | | |
| *Average Distance between/within groups*  Within level 'Poor' of factor 'OM' | | | | | | | | | | | | | |
|  | Control | | Acetone | | 0,01 | | 0,1 | | 1 | | 10 | | 100 |
| Control | 305.26 | |  | |  | |  | |  | |  | |  |
| Acetone | 315.5 | | 356.4 | |  | |  | |  | |  | |  |
| 1 | 372.03 | | 324.35 | | 256.82 | |  | |  | |  | |  |
| 10 | 381.85 | | 297 | | 212.84 | | 171.52 | |  | |  | |  |
| 100 | 444.18 | | 390.27 | | 254.33 | | 268.81 | | 286.42 | |  | |  |
| 1000 | 1078.8 | | 1204.7 | | 1387.6 | | 1397.1 | | 1478 | | 166.04 | |  |
| 10000 | 1101.9 | | 1228.9 | | 1409.2 | | 1422.9 | | 1497.4 | | 146.64 | | 144.87 |
| *PAIR-WISE TESTS*  Term 'CoxMO' for pairs of levels of factor 'OM' | | | | | | | | | | | | | |
| Within level 'Control' of factor 'Concentration' | | | | | | | | | | | | | |
| Groups | | | | Denominator | | | | | | Den. Df | | | |
| Rich x Poor | | | | 1*Res | | | | | | 9 | | | |
| *Average Distance between/within groups* | | | | | | | | | | | | | |
|  | | | | Rich | | | | | | Poor | | | |
| Rich | | | | 149.94 | | | | | |  | | | |
| Poor | | | | 1704.4 | | | | | | 305.26 | | | |
| Within level 'Acetone' of factor 'Concentration' | | | | | | | | | | | | | |
| Groups | | | | Denominator | | | | | | Den. Df | | | |
| Rich x Poor | | | | 1*Res | | | | | | 9 | | | |
| *Average Distance between/within groups* | | | | | | | | | | | | | |
|  | | | | Rico | | | | | | Pobre | | | |
| Rich | | | | 174.15 | | | | | |  | | | |
| Poor | | | | 1855.8 | | | | | | 356.4 | | | |
| Within level '1.0' of factor 'Concentration' | | | | | | | | | | | | | |
| Groups | | | | Denominator | | | | | | Den. Df | | | |
| Rich x Poor | | | | 1*Res | | | | | | 9 | | | |
| *Average Distance between/within groups* | | | | | | | | | | | | | |
|  | | | | Rich | | | | | | Poor | | | |
| Rich | | | | 228.5 | | | | | |  | | | |
| Poor | | | | 1901.6 | | | | | | 256.82 | | | |
| Within level '10.0' of factor 'Concentration' | | | | | | | | | | | | | |
| Groups | | | | Denominator | | | | | | Den. Df | | | |
| Rich x Poor | | | | 1*Res | | | | | | 8 | | | |
| *Average Distance between/within groups* | | | | | | | | | | | | | |
|  | | | | Rich | | | | | | Poor | | | |
| Rich | | | | 247.93 | | | | | |  | | | |
| Poor | | | | 1955.5 | | | | | | 171.52 | | | |
| Within level '100.0' of factor 'Concentration' | | | | | | | | | | | | | |
| Groups | | | | Denominator | | | | | | Den. Df | | | |
| Rich x Poor | | | | 1*Res | | | | | | 8 | | | |
| *Average Distance between/within groups* | | | | | | | | | | | | | |
|  | | | | Rich | | | | | | Poor | | | |
| Rich | | | | 251.44 | | | | | |  | | | |
| Poor | | | | 2135.8 | | | | | | 286.42 | | | |
| Within level '1000.0' of factor 'Concentration' | | | | | | | | | | | | | |
| Groups | | | | Denominator | | | | | | Den. Df | | | |
| Rich x Poor | | | | 1*Res | | | | | | 7 | | | |
| *Average Distance between/within groups* | | | | | | | | | | | | | |
|  | | | | Rich | | | | | | Poor | | | |
| Rich | | | | 159.47 | | | | | |  | | | |
| Poor | | | | 599.18 | | | | | | 166.04 | | | |
| Within level '10000,0' of factor 'Concentration' | | | | | | | | | | | | | |
| Groups | | | | Denominator | | | | | | Den. Df | | | |
| Rich x Poor | | | | 1*Res | | | | | | 10 | | | |
| *Average Distance between/within groups* | | | | | | | | | | | | | |
|  | | | | Rich | | | | | | Poor | | | |
| Rich | | | | 251.78 | | | | | |  | | | |
| Poor | | | | 517.11 | | | | | | 144.87 | | | |

Figure S3. Projection of the Euclidean distances of the replicates for the different concentrations of dichlofluanid, the control, and the analytical blank, considering all biomarkers analyzed in gill tissue. Figure S3a: Projection of replicates according to sediment organic matter contents. Figure S3b: Projection of replicates according to dichlofluanid concentrations.

Table S10. Results of PERMANOVA comparing sediment types (low and high OM). Analyses were based on Euclidean distances considering the biomarkers assessed in digestive glands.

|  | | | df | SS | | | MS | | | Pseudo-F | P(Perm) | | | Perms. | | |
| --- | --- | --- | --- | --- | --- | --- | --- | --- | --- | --- | --- | --- | --- | --- | --- | --- |
| Concentration | | | 6 | 5.5022E6 | | | 9.1704E5 | | | 18.605 | 0.001 | | | 999 | | |
| OM | | | 1 | 3.418E5 | | | 3.418E5 | | | 6.9345 | 0.01 | | | 999 | | |
| Co x OM | | | 6 | 5.5808E6 | | | 9.3013E5 | | | 18.871 | **0.001** | | | 999 | | |
| Residual | | | 62 | 3.056E6 | | | 49290 | | |  |  | | |  | | |
| Total | | | 75 | 1.6476E7 | | |  | | |  |  | | |  | | |
| Term 'CoxOM' for pairs of levels of factor 'Concentration'  Within level 'High' of factor 'OM' | | | | | | | | | | | | | | | | |
| Groups | | | | t | P(Perm) | | | Perms | | | | P(MC) | | |  |  |
| Acetone x Control | | | | 1.4047 | | 0.178 | | | 414 | | | | 0.187 | | |  |
| Acetone x 1 | | | | 0.39356 | | 0.728 | | | 56 | | | | 0.785 | | |  |
| Acetone x 10 | | | | 1.9114 | | 0.05 | | | 560 | | | | **0.048** | | |  |
| Acetone x 100 | | | | 2.3227 | | 0.022 | | | 412 | | | | **0.021** | | |  |
| Acetone x 1000 | | | | 1.701 | | 0.098 | | | 403 | | | | 0.12 | | |  |
| Acetone x 10000 | | | | 16.35 | | 0.002 | | | 574 | | | | **0.001** | | |  |
| Term 'CoxOM' for pairs of levels of factor 'Concentration'  Within level 'Low' of factor 'OM' | | | | | | | | | | | | | | | | |
| Groups | | | | t | P(Perm) | | | Perms | | | | P(MC) | | |  |  |
| Acetone x Control | | | | 3.0157 | | 0.015 | | | 126 | | | | **0.009** | | |  |
| Acetone x 1 | | | | 1.0786 | | 0.337 | | | 126 | | | | 0.333 | | |  |
| Acetone x 10 | | | | 1.2374 | | 0.22 | | | 126 | | | | 0.244 | | |  |
| Acetone x 100 | | | | 0.75066 | | 0.768 | | | 576 | | | | 0.49 | | |  |
| Acetone x 1000 | | | | 3.0765 | | 0.017 | | | 126 | | | | **0.005** | | |  |
| Acetone x 10000 | | | | 2.4867 | | 0.019 | | | 126 | | | | **0.018** | | |  |
| Term 'CoxOM' for pairs of levels of factor 'OM'  Factor 'Concentration' | | | | | | | | | | | | | | | | |
| Samples | | Groups | | t | P(Perm) | | | Perms | | | | P(MC) | | |  |  |
| Control | High x Low | | | 5.1064 | | 0.002 | | | 411 | | | | **0.001** | | |  |
| Acetone | High x Low | | | 3.1574 | | 0.039 | | | 126 | | | | **0.009** | | |  |
| 1 | High x Low | | | 3.267 | | 0.027 | | | 56 | | | | **0.005** | | |  |
| 10 | High x Low | | | 2.302 | | 0.027 | | | 573 | | | | **0.039** | | |  |
| 100 | High x Low | | | 0.45823 | | 0.927 | | | 770 | | | | 0.751 | | |  |
| 1000 | High x Low | | | 1.2048 | | 0.303 | | | 209 | | | | 0.269 | | |  |
| 10000 | High x Low | | | 16.063 | | 0.001 | | | 567 | | | | **0.001** | | |  |

**Table S11.** Additional results from PERMANOVA in the sediment bioassay with *Anomalocardia* *flexuosa*, for digestive glands.

| **Matrix: Sediment/ Tissue: Digestive gland / PERMANOVA/ Permutational MANOVA** | | | | | | | | | | | | | |
| --- | --- | --- | --- | --- | --- | --- | --- | --- | --- | --- | --- | --- | --- |
| *Resemblance worksheet*  Name: Resemblance  Data type: Distance  Selection: All  Resemblance: D1 Euclidean distance | | | | | | | | | | | | | |
| Sums of squares type: Type III (partial)  Fixed effects sum to zero for mixed terms  Permutation method: Permutation of residuals under a reduced model  Number of permutations: 999 | | | | | | | | | | | | | |
| Factors | | | Abbrev | | | | Type | | | | Levels | | |
| Concentration | | | Co | | | | Fixed | | | | 7 | | |
| OM | | | OM | | | | Fixed | | | | 2 | | |
| *Details of the expected mean squares (EMS) for the model* | | | | | | | | | | | | | |
| Source | | | | | | | EMS | | | | | | |
| Co | | | | | | | 1*V(Res) + 10.567*S(Co) | | | | | | |
| OM | | | | | | | 1*V(Res) + 36.137*S(MO) | | | | | | |
| Co x OM | | | | | | | 1*V(Res) + 5.2837*S(CoxMO) | | | | | | |
| Res | | | | | | | 1*V(Res) | | | | | | |
| *Construction of Pseudo-F ratio(s) from mean squares* | | | | | | | | | | | | | |
| Source | | Numerator | | | | Denominator | | Num.df | | | | Den.df | |
| Co | | 1*Co | | | | 1*Res | | 6 | | | | 62 | |
| OM | | 1*OM | | | | 1*Res | | 1 | | | | 62 | |
| Co x OM | | 1*Co x OM | | | | 1*Res | | 6 | | | | 62 | |
| *Estimates of components of variation* | | | | | | | | | | | | | |
| Source | | | | Estimate | | | | | | Sq.root | | | |
| S(Co) | | | | 82117 | | | | | | 286.56 | | | |
| S(MO) | | | | 8094.5 | | | | | | 89.97 | | | |
| S(CoxMO) | | | | 1.6671E5 | | | | | | 408.3 | | | |
| V(Res) | | | | 49290 | | | | | | 222.01 | | | |
| *PAIR-WISE TESTS*  Term 'CoxMO' for pairs of levels of factor 'Concentration'  Within level 'Rich' of factor 'OM' | | | | | | | | | | | | | |
| Groups | | | | Denominator | | | | | | Den.df | | | |
| Control x Acetone | | | | 1*Res | | | | | | 9 | | | |
| Acetone x 1 | | | | 1*Res | | | | | | 6 | | | |
| Acetone x 10 | | | | 1*Res | | | | | | 10 | | | |
| Acetone x 100 | | | | 1*Res | | | | | | 9 | | | |
| Acetone x 1000 | | | | 1*Res | | | | | | 9 | | | |
| Acetone x 10000 | | | | 1*Res | | | | | | 10 | | | |
| *Average Distance between/within groups*  Within level 'Rich' of factor 'OM' | | | | | | | | | | | | | |
|  | Control | | Acetone | | 0,01 | | 0,1 | | 1 | | 10 | | 100 |
| Control | 106.94 | |  | |  | |  | |  | |  | |  |
| Acetone | 129.36 | | 141.64 | |  | |  | |  | |  | |  |
| 1 | 149.62 | | 128.92 | | 167.99 | |  | |  | |  | |  |
| 10 | 195.14 | | 191.98 | | 176.09 | | 161.74 | |  | |  | |  |
| 100 | 209.58 | | 186.38 | | 168.36 | | 149.97 | | 119.71 | |  | |  |
| 1000 | 537.98 | | 574.55 | | 568.36 | | 511.48 | | 539.24 | | 721.27 | |  |
| 10000 | 1423.6 | | 1501.5 | | 1509.3 | | 1461.6 | | 1522.8 | | 1070.8 | | 219.46 |
| *PAIR-WISE TESTS*  Term 'CoxMO' for pairs of levels of factor 'Concentration'  Within level 'Poor' of factor 'OM' | | | | | | | | | | | | | |
| Groups | | | | Denominator | | | | | | Den.df | | | |
| Control x Acetone | | | | 1*Res | | | | | | 8 | | | |
| Acetona x 1 | | | | 1*Res | | | | | | 8 | | | |
| Acetona x 10 | | | | 1*Res | | | | | | 8 | | | |
| Acetona x 100 | | | | 1*Res | | | | | | 10 | | | |
| Acetona x 1000 | | | | 1*Res | | | | | | 7 | | | |
| Acetona x 10000 | | | | 1*Res | | | | | | 8 | | | |
| *Average Distance between/within groups*  Within level 'Poor' of factor 'OM' | | | | | | | | | | | | | |
|  | Control | | Acetone | | 0,01 | | 0,1 | | 1 | | 10 | | 100 |
| Control | 57.23 | |  | |  | |  | |  | |  | |  |
| Acetone | 139.43 | | 102.24 | |  | |  | |  | |  | |  |
| 1 | 105.79 | | 94.062 | | 90.786 | |  | |  | |  | |  |
| 10 | 135.77 | | 90.327 | | 79.603 | | 72.875 | |  | |  | |  |
| 100 | 283.8 | | 224.34 | | 233.64 | | 218.47 | | 352.27 | |  | |  |
| 1000 | 182.99 | | 151.15 | | 178.61 | | 194.17 | | 273.67 | | 56.194 | |  |
| 10000 | 137.25 | | 125.99 | | 139.36 | | 156.96 | | 260.38 | | 68.573 | | 60.747 |
| *PAIR-WISE TESTS*  Term 'CoxMO' for pairs of levels of factor 'OM' | | | | | | | | | | | | | |
| Within level 'Control' of factor 'Concentration' | | | | | | | | | | | | | |
| Groups | | | | Denominator | | | | | | Den. Df | | | |
| Rich x Poor | | | | 1*Res | | | | | | 9 | | | |
| *Average Distance between/within groups* | | | | | | | | | | | | | |
|  | | | | Rich | | | | | | Poot | | | |
| Rich | | | | 106.94 | | | | | |  | | | |
| Poor | | | | 215.01 | | | | | | 57.23 | | | |
| Within level 'Acetone' of factor 'Concentration' | | | | | | | | | | | | | |
| Groups | | | | Denominator | | | | | | Den. Df | | | |
| Rich x Poor | | | | 1*Res | | | | | | 8 | | | |
| *Average Distance between/within groups* | | | | | | | | | | | | | |
|  | | | | Rich | | | | | | Poor | | | |
| Rich | | | | 141.64 | | | | | |  | | | |
| Poor | | | | 208.79 | | | | | | 102.24 | | | |
| Within level '1' of factor 'Concentration' | | | | | | | | | | | | | |
| Groups | | | | Denominator | | | | | | Den. Df | | | |
| Rich x Poor | | | | 1*Res | | | | | | 6 | | | |
| *Average Distance between/within groups* | | | | | | | | | | | | | |
|  | | | | Rich | | | | | | Poor | | | |
| Rich | | | | 167.99 | | | | | |  | | | |
| Poor | | | | 251.26 | | | | | | 90.786 | | | |
| Within level '10' of factor 'Concentration' | | | | | | | | | | | | | |
| Groups | | | | Denominator | | | | | | Den. Df | | | |
| Rich x Poor | | | | 1*Res | | | | | | 10 | | | |
| *Average Distance between/within groups* | | | | | | | | | | | | | |
|  | | | | Rich | | | | | | Poor | | | |
| Rich | | | | 161.74 | | | | | |  | | | |
| Poor | | | | 164.25 | | | | | | 72.875 | | | |
| Within level '100' of factor 'Concentration' | | | | | | | | | | | | | |
| Groups | | | | Denominator | | | | | | Den. Df | | | |
| Rich x Poor | | | | 1*Res | | | | | | 11 | | | |
| *Average Distance between/within groups* | | | | | | | | | | | | | |
|  | | | | Rich | | | | | | Poor | | | |
| Rich | | | | 119.71 | | | | | |  | | | |
| Poor | | | | 274.09 | | | | | | 352.27 | | | |
| Within level '1000' of factor 'Concentration' | | | | | | | | | | | | | |
| Groups | | | | Denominator | | | | | | Den. Df | | | |
| Rich x Poor | | | | 1*Res | | | | | | 8 | | | |
| *Average Distance between/within groups* | | | | | | | | | | | | | |
|  | | | | Rich | | | | | | Poor | | | |
| Rich | | | | 721.27 | | | | | |  | | | |
| Poor | | | | 518.98 | | | | | | 56.194 | | | |
| Within level '10000' of factor 'Concentration' | | | | | | | | | | | | | |
| Groups | | | | Denominator | | | | | | Den. Df | | | |
| Rich x Poor | | | | 1*Res | | | | | | 10 | | | |
| *Average Distance between/within groups* | | | | | | | | | | | | | |
|  | | | | Rich | | | | | | Poor | | | |
| Rich | | | | 219.46 | | | | | |  | | | |
| Poor | | | | 1350.8 | | | | | | 60.747 | | | |

Fig S4. Projection of Euclidean distances for the replicates exposed to different concentrations of dichlofluanid, the control, and the solvent control, based on all biomarkers analyzed in digestive gland tissue. Figure S4a: Projection of replicates according to dichlofluanid concentrations. Figure 11b: Projection of replicates according to the organic matter content of the sediments.
